# Investigations on recyclisation and hydrolysis in avibactam mediated serine β-lactamase inhibition†
†Electronic supplementary information (ESI) available. See DOI: 10.1039/c6ob00353b



**DOI:** 10.1039/c6ob00353b

**Published:** 2016-04-13

**Authors:** Hwanho Choi, Robert S. Paton, Hwangseo Park, Christopher J. Schofield

**Affiliations:** a Department of Bioscience and Biotechnology , Sejong University , 209 Neungdong-ro , Kwangjin-gu , Seoul 143-747 , Korea . Email: hspark@sejong.ac.kr ; Email: christopher.schofield@chem.ox.ac.uk ; Fax: +82 2 3408 3334, +44 (0)1865 285 022 ; Tel: +82 2 3408 3766, +44 (0)1865 275 625; b Chemical Research Laboratory , University of Oxford , Mansfield Road , Oxford OX1 3TA , UK

## Abstract

β-Lactams inhibit penicillin-binding proteins (PBPs) and serine β-lactamases by acylation of a nucleophilic active site serine. Avibactam is approved for clinical use in combination with ceftazidime, and is a breakthrough non β-lactam β-lactamase inhibitor also reacting *via* serine acylation. Molecular dynamics (MD) and quantum chemical calculations on avibactam-mediated inhibition of a clinically relevant cephalosporinase reveal that recyclisation of the avibactam derived carbamoyl complex is favoured over hydrolysis. In contrast, we show that analogous recyclisation in β-lactam mediated inhibition is disfavoured. Avibactam recyclisation is promoted by a proton shuttle, a ‘structural’ water protonating the nucleophilic serine, and stabilization of the negative charge developed on aminocarbonyl oxygen. The results imply the potential of calculations for distinguishing between bifurcating pathways during inhibition and in generating hypotheses for predicting resistance. The inability of β-lactams to undergo recyclisation may be an Achilles heel, but one that can be addressed by suitably functionalized reversibly binding inhibitors.

## Abstract 2

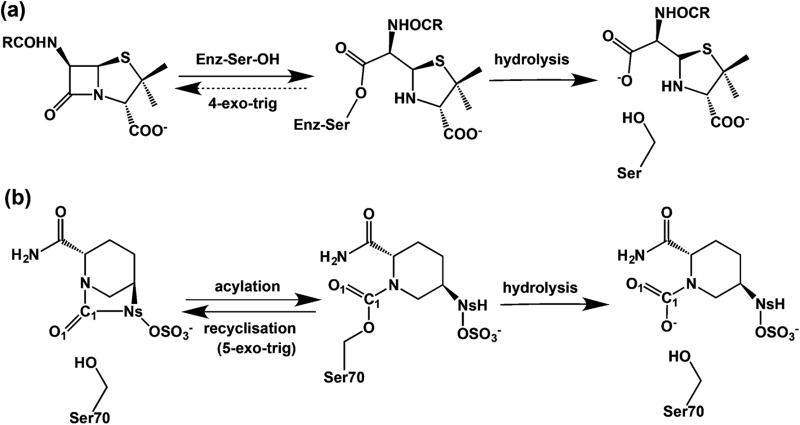
In contrast to the β-lactams, which react irreversibly, avibactam reacts reversibly with serine β-lactamases.

## Introduction

β-Lactam containing antibacterials, which remain amongst the most important of all pharmaceuticals,^1^ target transpeptidases/carboxypeptidases or penicillin binding proteins (PBPs) involved in bacterial cell wall biosynthesis by a mechanism involving acylation of a nucleophilic serine-residue.^2–5^ The progenitor penicillins were followed by successive β-lactam generations, including the cephalosporins, monobactams, and carbapenems, work driven in part by a desire to combat resistance due to β-lactamases, which catalyze β-lactam hydrolysis to give inactive β-amino acids.^6^ An alternative strategy is to combine a β-lactam antibacterial with a β-lactamase inhibitor. Three such β-lactamase inhibitors (clavulanic acid, tazobactam, and sulbactam)^7,8^ protect against class A serine β-lactamases (‘penicillinases’), but do not protect against other β-lactamase classes,^9^ notably class C cephalosporinases. The serine β-lactamases (SBLs) operate *via* mechanisms related to those of the penicillin binding proteins (PBPs). A key difference is that the acyl-enzyme complexes (AECs) formed by reaction of SBLs with β-lactam substrates are more hydrolytically labile than those of PBPs.^10,11^ Clinically useful SBL inhibitors form hydrolysis resistant acyl-enzymes, in part because they provide a sink that traps the ‘hydrolytic’ water. The dominance of β-lactam compounds as useful PBP/β-lactamase inhibitors led to the proposal they are sacrosanct in this regard.^12^

**Fig. 1:**
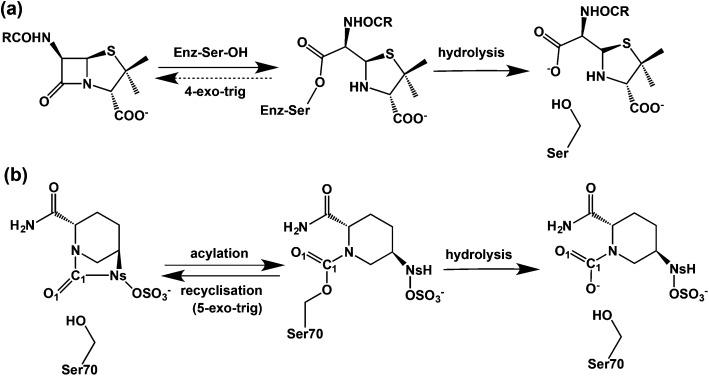
β-Lactams react irreversibly with penicillin binding proteins/β-lactamases nucleophilic enzymes to form an acyl-enzyme complex in contrast to avibactam which reacts reversibly. (a) Irreversible reaction of a β-lactam with nucleophilic serine enzyme as exemplified by reaction of a penicillin with a penicillin binding protein (transpeptidase) to give a stable acyl-enzyme complex, which only undergoes slow hydrolysis; (b) schematic representation of the acylation, recyclisation, and hydrolysis reactions between a serine β-lactamase and avibactam.

Attempts to replace β-lactams began early,^13^ with synthesis of γ-lactam and other analogues.^14^ Successes were achieved when unsaturated bicyclic γ-lactams were found to be antibacterials.^14^ The isolation of the cycloserine containing natural product lactivicin, further revealed potential for non-β-lactam acylating agents.^15,20,42^ Model studies led to the idea that an advantage of β-lactam inhibition is effectively irreversible acylation. In contrast inhibition by 5- or 6-membered lactams is compromised by kinetically favoured reaction of the acylated-enzyme to reform the lactam (Fig. 1).^16–18^ this reversibility can be countered by features that hinder recyclisation, *e.g.* ring strain, steric, or electronic factors.^19–21^

Avibactam ((2*S*,5*R*)-7-oxo-6-(sulfoxy)-1,6-diazabicyclo[3,2,1]octane-2-carboxamide) is a breakthrough, because it inhibits class A, C, and some class D enzymes and is the first non-β-lactam β-lactamase inhibitor to complete clinical trials.^22–25^ Avibactam has an unusual bicyclic structure comprising a strained cyclic urea that was developed to enable efficient acylation by optimised interaction with the active site.^26,27^ Avibactam potently inhibits β-lactamases including the class A extended-spectrum β-lactamase (CTX-M-15) and carbapenemase (KPC-2), and class C AmpC enzymes.^28,29^

Avibactam inhibits serine β-lactamases *via* reaction of its urea carbonyl with the nucleophilic serine (Fig. 1).^30,31^ In contrast to lactivicin^32^ and β-lactam mediated acylation,^8^ avibactam mediated acylation is reversible (Fig. 1(b)).^27^ Hydrolysis of the avibactam derived acyl-enzyme can occur, likely *via* initial loss of the sulfate, as shown for the KPC-2 β-lactamase, where hydrolysis is faster than for CTX-M-15.^33^ Understanding factors regulating the balance between reversibility/irreversibility and recylisation/hydrolysis of the inhibitory complexes is important because hydrolysis of cyclic urea of avibactam, like β-lactams, is irreversible and hence inactivating.

Structures for avibactam-acylated SBLs are reported.^34–36^ In that for the CTX-M-15 avibactam complex,^34^ the nitrogen of the hydroxylamine-*O*-sulfonate group of avibactam and a structural water (Wat411) are close to the carbamoyl group linking to the nucleophilic Ser70, and are apparently stabilized by the interactions with the Lys73-Ser130 and Glu166-Asn170 dyads, respectively. Although the structures suggest recyclisation to avibactam is feasible, they do not inform on parameters determining its rate relative to hydrolysis.^36^

Computational studies have been carried out on acylation of some β-lactamases by β-lactam antibiotics/inhibitors, including on acylation from the non-covalent complex formed between class C β-lactamases and β-lactams;^37^ combined with experimental work residues playing roles in acid/base catalysis have been identified. Sgrignani *et al.* recently addressed the mechanism of the formation of the carbamoyl-complex between the TEM-1 class A β-lactamase TEM-1 and avibactam.^38^ The results of hybrid quantum chemical/molecular mechanics simulations indicated that the rate-limiting process in acylation was water-assisted deprotonation of Glu166 by the nucleophilic residue Ser70 in order to form a tetrahedral intermediate. It was also concluded that the Nε-amino group of Lys73 plays a key role in providing acid base catalysis, including *via* proton transfer to Ser-130, which in turn protonates the avibactam derived urea nitrogens.^38^

The scope of previous computational investigations of the reaction between β-lactamases and ligands has been limited to possible mechanisms of covalent complex formation. The key mechanistic question of recyclisation has not been addressed even for β-lactams (non-enzymatic β-lactam synthesis has been studied by calculations.^39^). We report the results of MD simulations and quantum chemical calculations on the recyclisation and hydrolysis of the avibactam derived acyl-enzyme complex with a clinically important SBL in comparison with those for a β-lactam (oxacillin). On the basis of the structural features of transition states and reaction intermediates identified, we address the molecular driving forces that render recyclisation of avibactam from the acyl-enzyme complex possible. The results will be useful in the design of new β-lactamase inhibitors following avibactam and in predicting the properties of clinically observed substitutions of β-lactamases as they emerge in response to use of avibactam.

## Results and discussion

### MD simulation studies on the Glu166 protonation state in the CTX-M-15 complex with avibactam

**Fig. 2:**
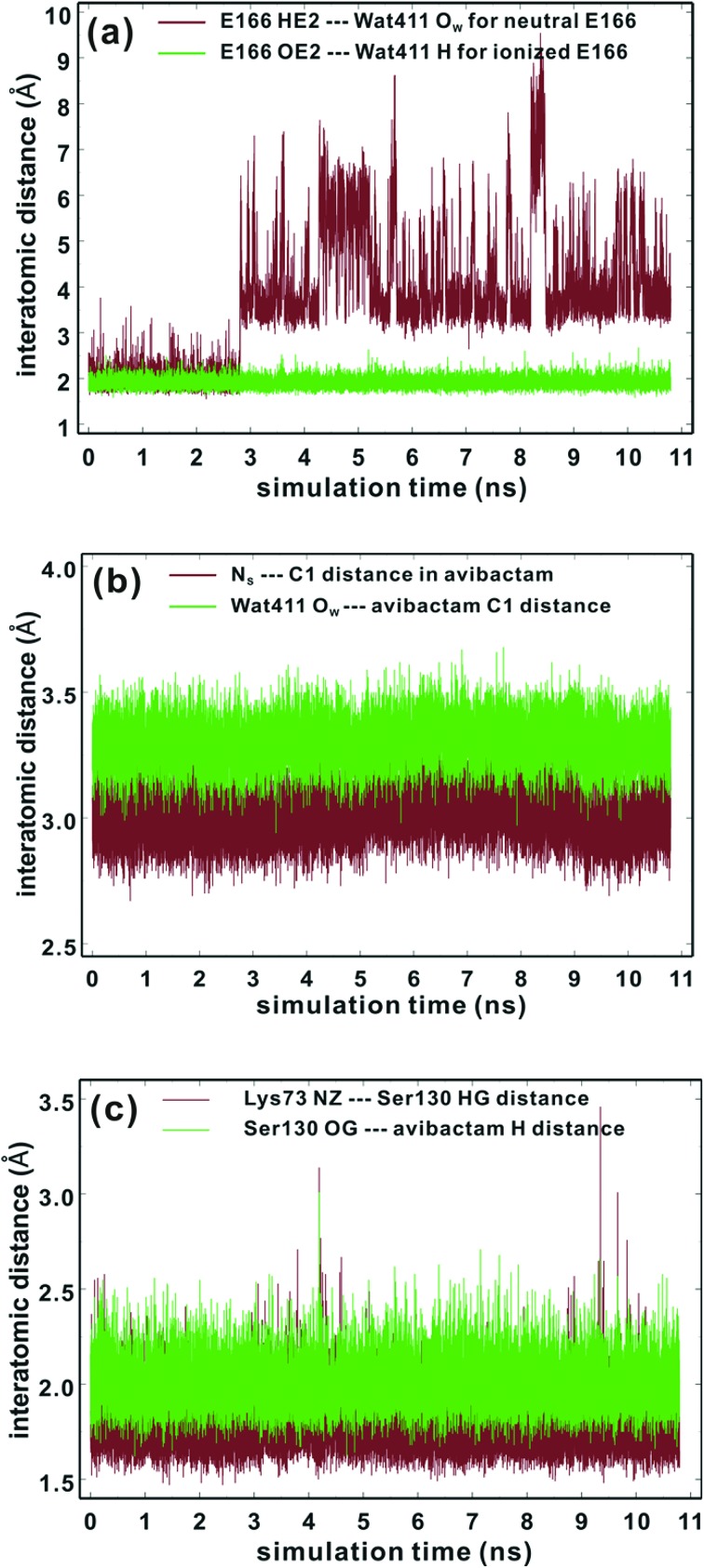
Time evolutions of the selected interatomic distances during MD simulations of the CTX-M-15 avibactam complex in the explicit water model. See text and Fig. 6 for identification of atoms. (a) Hydrogen-bond distances between neutral Glu166 and Wat411 (brown) and those between ionized Glu166 and Wat411 (green). (b) Interatomic distances between N_S_ and C1 atoms of avibactam (brown) and those between Wat411 O_W_ and C1 of avibactam (green). (c) Hydrogen bond distances between Lys73 and Ser130 (brown) and those between Ser130 and avibactam (green).

To investigate the question of reversible reaction of avibactam with its targets we focused on the clinically important class A extended-spectrum β-lactamase CTX-M-15 for which structures are available (PDB entry: 4HBU).^34^ Crystallographic analyses suggest Wat411, positioned to hydrogen bond with Glu166, has a role in stabilizing the covalent CTX-M-15-avibactam acyl-enzyme complex (henceforth avibactam complex) (Fig. S1†).^34^ First, we carried out MD simulations to investigate the maintenance of the complex structure and the Glu166 protonation state. The results indicate that the CTX-M-15 avibactam complex is conformationally stable irrespective of Glu166 protonation (Fig. S2†). Studies on the time dependences of the hydrogen bond distance of Wat411 reveal that when Glu166 is deprotonated, the hydrogen bond is sustained for 98% of simulation time (Fig. 2a), when a distance limit for the O···H hydrogen bond is set at 2.2 Å.^40^ The time-averaged distance between Glu166 OE2 and the Wat411 oxygen (O_W_) is 2.68 Å; this is close to the reported refined distance of 2.60 Å in the CTX-M-15-avibactam structure.^34^ In contrast, when Glu166 is in its neutral form, HE2 of Glu166 is positioned away from Wat411 O_W_, at a distance >3.0 Å for most of the simulation time after 2.81 ns. Similar dynamic behavior is observed in the distances between Glu166 OE2 and Wat411 O_W_ with protonated Glu166. These results imply the crystallographically observed hydrogen bond between Glu166 and Wat411 is stable in the CTX-M-15 avibactam complex when Glu166 is deprotonated; note that this contrasts with the interpretation of the results of crystallographic studies on the CTX-M-15 avibactam complex.^34^ We thus used the deprotonated form of Glu166 in subsequent investigations on recyclisation and hydrolysis of the CTX-M-15-avibactam complex.

### MD simulations on the possibility of recyclisation of the β-lactamase-avibactam complex

Kinetic studies indicate that the avibactam complex preferentially reforms avibactam rather than undergo hydrolysis (both these rates are slower than for formation of the avibactam complex).^27,34^ To investigate the molecular basis of this, we compared the time evolution of the distance between O_W_ of Wat411 and the carbonyl carbon of ring-opened avibactam (C1) to that between the nitrogen of the avibactam derived hydroxylamine-*O*-sulfonate group (N_S_) and C1 atom (henceforth N_S_ and C1) (Fig. 2b). The intramolecular N_S_···C1 distances in avibactam were closer than the intermolecular O_W_···C1 distances for >99% of simulation time, consistent with recyclisation being preferred over hydrolysis.

In order for ‘deacylation’ of Ser70 in the avibactam-complex to occur *via* an addition–elimination reaction, a stereoelectronically acceptable relationship between the nucleophile (N_S_ or O_W_) and C1 carbonyl must be established^41^ (‘near attack conformer, NAC’^43^). The N_S_···C1 (*i.e.* the avibactam urea derived nitrogen and carbonyl) distance falls within the 3.2 Å (the proposed distance limit for NAC involving nucleophilic attack on a carbonyl)^43^ for 97% of the MD simulation time, compared to only 14% for the O_W_···C1 distance, again supporting recyclisation over hydrolysis. The angle formed between N_S_ and central carbonyl group (N_S_···C1=O angle) ranges from 100 to 110° for 82% of residence time. These results indicate most of the MD trajectory snapshots of CTX-M-15-avibactam complex fall within criteria favourable for nucleophilic addition.^41,44^ Note that, since MD simulations do not account for stereoelectronic effects, torsional and non-bonding interactions can likely generate arrangements favourable for ring closure.

**Fig. 3:**
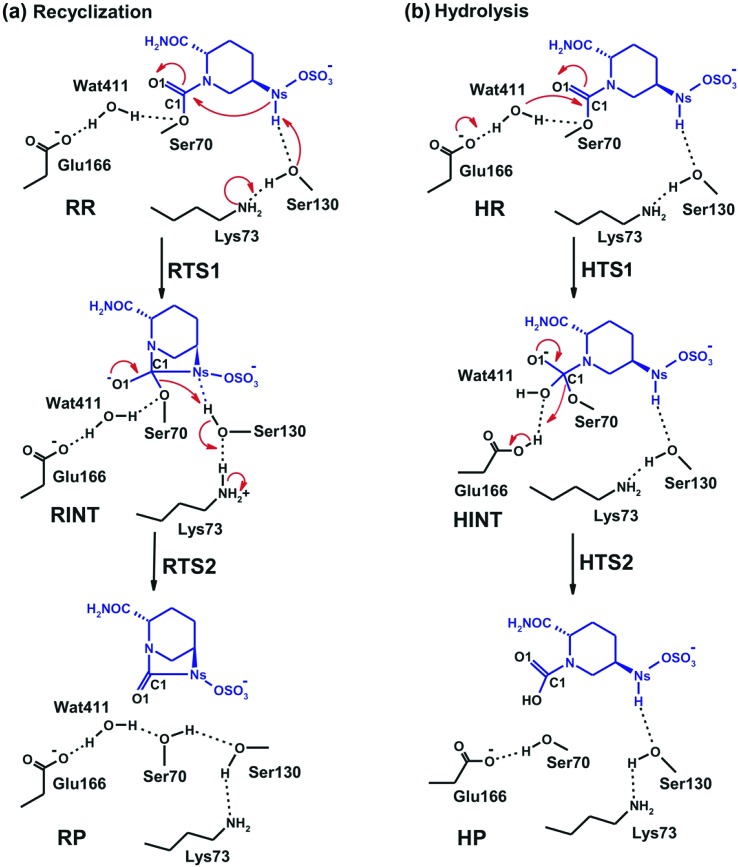
Possible fates for reaction of the CTX-M-15 avibactam covalent complex: (a) recyclisation, and (b) hydrolysis. **RR** and **HR**, **RTS1** and **HTS1**, **RINT** and **HINT**, **RTS2** and **HTS2**, and **RP** and **HP** denote the reactive complex, first transition state, reaction intermediate, second transition state, and final energy minimum, respectively, during the recyclisation and hydrolysis reactions, respectively.

According to X-ray analysis of the CTX-M-15 avibactam complex,^34^ consecutive proton transfer processes (one from Ser130 to Lys73, and the other from Ns(avibactam) to Ser130) are required for recyclisation (similar processes occur during formation of the avibactam complex^38^). We thus investigated the dynamic stabilities of the hydrogen bonds between Ser130 and Lys73 and between the avibactam –N_S_H– group and Ser130 (Fig. 2c). The Ser130 HG···Lys73 NZ and avibactam N_S_H···Ser130 OG hydrogen bonds are present for 99% and 93% of simulation time, respectively, with the respective time-averaged distances of 1.78 and 2.00 Å. Note the hydrogen bond between Lys73 and Ser130 is unusually strong in some trajectory snapshots, with the associated N···H distance being close to 1.5 Å, supporting the possibility of proton transfer from Ser130 to Lys73, which would in turn will facilitate nucleophilic addition to C1 of the covalent complex by deprotonating N_S_. These results further support recyclisation over carbamate hydrolysis. However, hydrolysis of the avibactam complex is of interest with respect to evolution of resistance, especially given that Wat411 is proximate to C1 in some trajectory snapshots (Fig. 2b) in which the nucleophilic attack may be possible. Therefore, we examined the two possibilities of reaction for the CTX-M-1 avibactam complex (Fig. 3) using quantum chemical calculations at the MP2/6-31G*//RHF/6-31G* level.

### Avibactam recyclisation in the active site of CTX-M-15

**Fig. 4:**
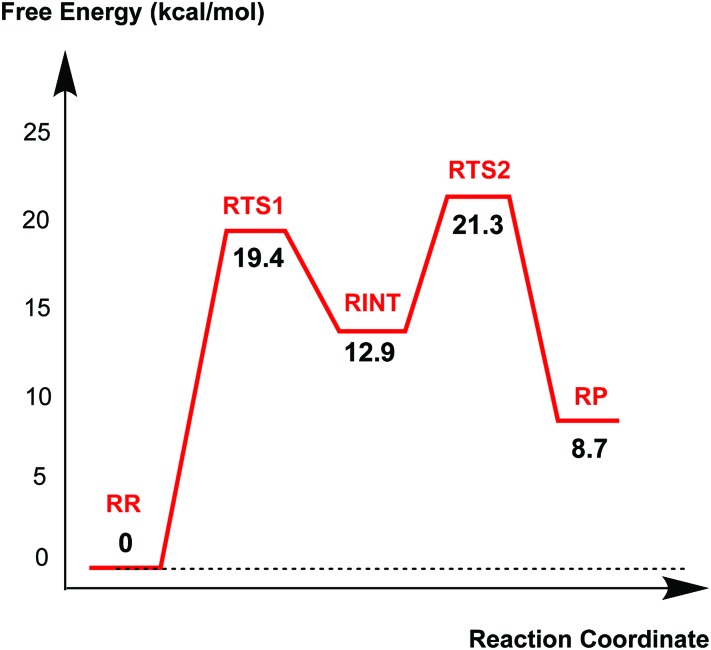
Free energy profile of the intrinsic reaction coordinate for the recyclisation to give avibactam from the covalent complex with CTX-M-15. Free energy is measured from reactant complex (**RR**) for each stationary-state structure using the MP2/6-31G* level of calculation. **RTS1**, **RINT**, **RTS2**, and **RP** represent the first transition state, reaction intermediate, second transition state, and final energy minimum, respectively, during the recyclisation reaction.

Fig. 4 displays the results of MP2/6-31G* calculations on the recyclisation to avibactam. The first step involves intramolecular nucleophilic attack of N_S_ on C1, accompanied by proton abstraction from N_S_ by Ser130 (Fig. 3). The tetrahedral reaction intermediate (**RINT**) forms with a calculated free energy of 12.9 kcal mol^–1^ above the reactant complex (**RR**) *via* the first transition state (**RTS1**); the calculated free energy of activation of which is 19.4 kcal mol^–1^. The OG atom of Ser70 moves away from C1 in the second step to form a non-covalent avibactam complex (**RP**). The activation barrier for this reaction step is estimated to be 8.4 kcal mol^–1^. The calculated overall free energy of activation for recyclisation amounts to 21.3 kcal mol^–1^ (from **RR** to **RTS2**). Although care should be taken in comparing absolute values, due the different systems/methods involved, it is interesting to compare the calculated energetics for recyclisation to avibactam with those for the ring-opening reaction to form the covalent complex with the TEM-1 β-lactamase.^38^ In our calculations at the MP2/6-31G*//RHF/6-31G* level, the activation barrier for the formation of tetrahedral intermediate (**RP** → **RINT**) is 6.1 kcal mol^–1^ higher than that for cleaving the C1–N_S_ bond (**RINT** → **RR**). This is consistent with the previous hybrid QM/MM investigations on the inhibition of TEM-1 enzyme by avibactam in which deprotonation of Ser70 to attack the C1 atom in order to form the tetrahedral intermediate was found to be rate-limiting.^38^

**Fig. 5:**
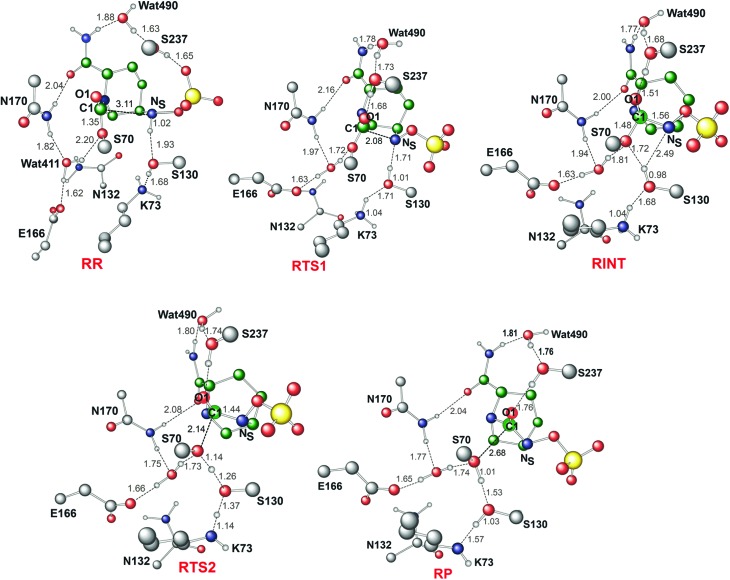
Calculated structures of the energy minima and transition states for the recyclisation of the avibactam complex in the CTX-M-15 active site. Selected interatomic distances are in Å. **RR**, **RTS1**, **RINT**, **RTS2**, and **RP** represent the reactive complex, first transition state, reaction intermediate, second transition state, and final energy minimum, respectively, during the recyclisation reaction.

We now detail the structural changes occurring during recyclisation (Fig. 3 and 5). In the starting point for recyclisation (**RR**), which was obtained with full geometry optimization, N_S_ of avibactam is close to C1 (3.11 Å) with the N_S_···C1=O angle of 109°, *i.e.* within the Bürgi-Dunitz criteria.^43,44^ In **RR**, however, Wat411 also remains in the vicinity of the carbamoyl group being positioned to make hydrogen bonds with the side chains of Glu166, Asn170, and Ser70. Overall, **RR** retains the structural features observed in the crystal structure of CTX-M-15 avibactam complex^32^ and those in MD simulations in aqueous solution (data not shown because of similarity to the crystal structure).

The avibactam recyclisation reaction starts from **RR** with approach of N_S_ toward C1 (Fig. 5). When the N_S_···C1 interatomic distance falls to 2.08 Å, the reaction reaches **RTS1**; nucleophilic addition is facilitated by deprotonation of N_S_ by the Ser130 hydroxyl, which is in turn deprotonated by the neutral side chain of Lys73. The transition state **RTS1** is late in terms of both C1–N_S_ bond formation and proton transfer from N_S_ to Ser130, corresponding to ∼65% and 95% towards formation of **RINT**, respectively. The single imaginary frequency of **RTS1** is dominated by the bond forming motions of C1–N_S_ bond in avibactam and OG–HG bond in Ser130, implying the activation free energy of this step arises substantially from distortion of planar geometry around the acyl-enzyme complex carbonyl due to the nucleophilic attack of deprotonated N_S_. Concomitant with approach of N_S_ toward C1, the Ser237 hydroxyl moves towards the carbonyl oxygen (O1) of the carbamoyl group to avoid collision with the avibactam-derived sulfate. This movement establishes a strong hydrogen bond between Ser237 and O1 in **RTS1** and **RINT**, which stabilises the developing negative charge on O1 due to the addition of N_S_ to C1. Simultaneously with reaction from **RR** to **RTS1**, the hydrogen bond between Wat411 and Ser70 is strengthened (decreasing from 2.20 to 1.72 Å). Based on the apparent maintenance of the Wat490···Ser237, Wat411···Glu166, and Asn170···Wat411 hydrogen bonds in going from **RR** to **RTS1**, it is likely that Wat490, Glu166 and Asn170 play roles in positioning Ser237 and Wat411, respectively.

Further approach of N_S_ to C1 to form the C1–N_S_ bond leads to formation of a ‘tetrahedral’ sp^3^ hybridised intermediate (**RINT**). Concomitant with this reaction, the Ser237···O1 hydrogen bond strengthens due to the accumulation of negative charge on O1. Interestingly, the Ser130 hydroxyl group also moves from N_S_ to Ser70 to serve as a hydrogen bond donor (as does Wat411). These two hydrogen bonds appear to promote cleavage of Ser70–C1 bond by stabilising the ‘leaving’ OG atom of Ser70.

The second step in recyclisation is initiated by elongation of the Ser70–C1 bond in **RINT**, accompanied by proton transfer from OG of Ser130 to Ser70. This proton transfer is enabled by the protonation of Ser130 by the positively charged Lys73 side-chain. As a consequence of the two consecutive proton-transfer processes, the Ser70–C1 distance increases from 1.48 Å in **RINT** to 2.14 Å in **RTS2**. A significant feature associated with **RTS2** is that the two hydrogens attached to Ser130 and Lys73 are asymmetrically shared by the donor and acceptor atoms. This result differs from that of **RTS1** in which the two protons are fully transferred to the acceptor atoms. Overall, the results predict that **RTS2** is intermediate and ‘late’ in terms of the rupture of the Ser70–C1 bond and the formation of O–H bond in Ser70, respectively, which exhibit 55% and 82% advancement to complete the second step of recyclisation.

From **RTS2**, a further increase in the Ser70···C1 distance carries the reaction system to the final minimum energy structure (**RP**), *i.e.* non-covalently bound avibactam in the active site (Fig. 5). Planarity around C1 is restored in **RP** due to the cleavage of Ser70–C1 bond. Simultaneously, proton transfer processes involving protonated Ser70, Ser130, and Lys73 are completed to stabilize the leaving group. Wat411 maintains a hydrogen bond with Ser70 throughout the reaction, consistent with its role in enabling ‘release’ of Ser70 from the acyl-enzyme complex. The role of the Lys73-Ser130 dyad appears to be crucial in recyclisation (as in formation of the avibactam complex^38^). It acts as a general base to activate N_S_ as a nucleophile in the first step and as a general acid to stabilize the negative charge of the leaving Ser70 in the second step.

### Mechanism of hydrolysis in the CTX-M-15 active site

**Fig. 6:**
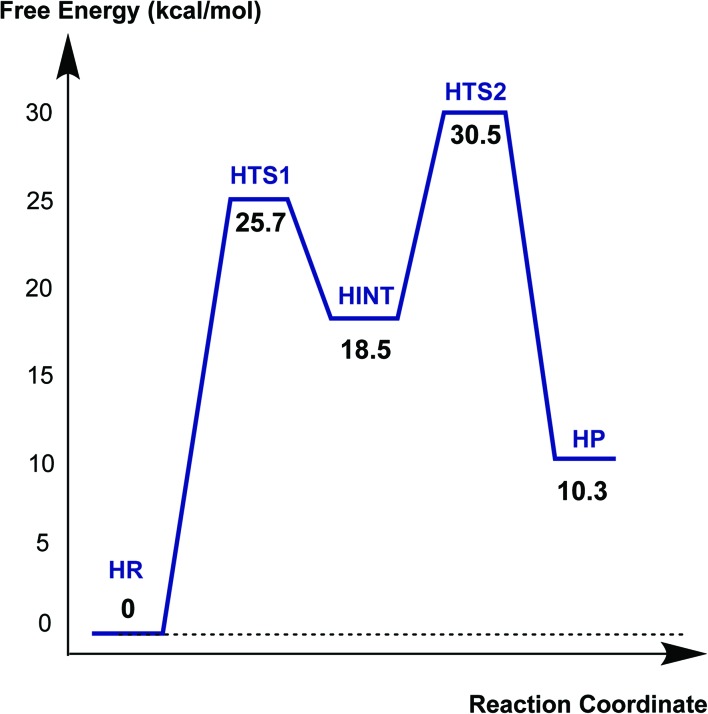
Free energy profile of the intrinsic reaction coordinate for hydrolysis of the Ser70-avibactam bond in the CTX-M-15 active site. Free energy is measured from reactant complex (**HR**) for each stationary-state structure using the MP2/6-31G* level of calculations. **HTS1**, **HINT**, **HTS2**, and **HP** represent the first transition state, reaction intermediate, second transition state, and final energy minimum, respectively, during the hydrolysis reaction.

Fig. 6 displays the calculated free energy profile for hydrolysis of the carbamoyl group linking Ser70 and avibactam. The first activation barrier given by the free energy difference between the reactive complex (**HR**) and the first transition state (**HTS1**) is 6.3 kcal mol^–1^ higher than that for recyclisation. Similarly, the barrier for the formation of the tetrahedral reaction intermediate (**HINT**) increases by 5.6 kcal mol^–1^ when compared to that in recyclisation. Consistent with experimental observations,^27^ hydrolysis is calculated to be much less efficient than recyclisation. From **HINT**, an additional free energy barrier of 12.0 kcal mol^–1^ must be surmounted to reach the rate-limiting second transition state (**HTS2**) for the hydrolytic cleavage of Ser70–C1 in the acyl-enzyme complex. The calculated overall free energy of activation is 30.5 kcal mol^–1^. Importantly, this is substantially higher than for recyclisation (by 9.2 kcal mol^–1^). These results clearly indicate a preference for recyclisation over hydrolysis, consistent with the reversibility observed in kinetic studies.^27^ In contrast to the large difference in free energies of activation, the calculated free energy change for hydrolysis appears to be higher than that of the recyclisation by only 1.6 kcal mol^–1^. Thus, kinetic factors are important in determining the fate of the CTX-M-15 avibactam complex.

**Fig. 7:**
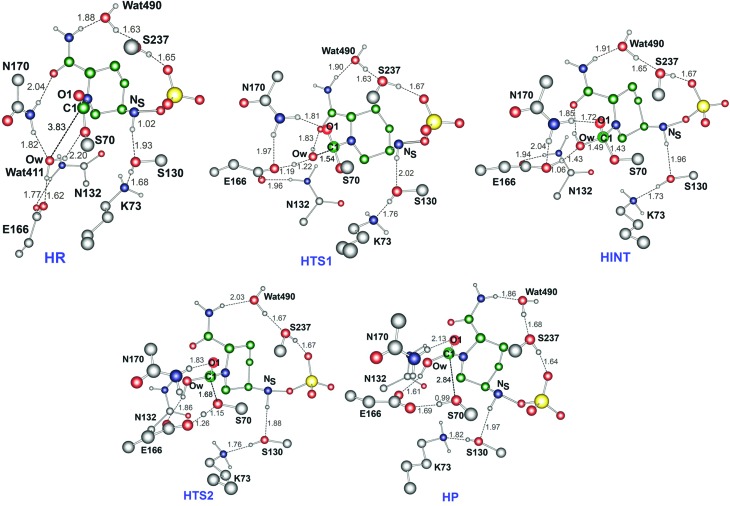
Calculated structures of the energy minima and transition states for hydrolysis of the avibactam derived acyl-enzyme complex in the CTX-M-15 active site. Selected interatomic distances are given in Å. **HR**, **HTS1**, **HINT**, **HTS2**, and **HP** represent the reactive complex, first transition state, reaction intermediate, second transition state, and final energy minimum, respectively, during the hydrolysis reaction.

Fig. 7 displays the structures of the reacting system at the energy minima and transition states identified on the intrinsic reaction coordinate (IRC) for hydrolysis. In the reactive complex **HR** (identical to **RR** in Fig. 5), Wat411 resides 3.83 Å distant from C1. The reaction proceeds from **HR** as the O_W_ atom of Wat411 approaches C1 in an approximately perpendicular manner to the plane of the carbamoyl group of the covalent complex. When the O_W_···C1 distance reduces to 1.54 Å, the reaction system reaches **HTS1** in which C1 has undergone significant pyramidalization: the average of the four bond angles around C1 is 108° in **HTS1** as compared to 120° in **HR**. Nucleophilic addition is likely facilitated by the deprotonation of Wat411 by Glu166, as reflected by a decrease in the hydrogen bond distance from 1.62 Å in **HR** to 1.19 Å in **HTS1**. This partial deprotonation apparently causes the second hydrogen of Wat411 to ‘flip’ away from Ser70 towards the terminal aminocarbonyl oxygen of avibactam, leading to a new O–H···O hydrogen bond. This may have the effect of destabilizing **HTS1** due to the loss of a hydrogen bond around the reaction center. In **HTS1**, the role of general acid catalyst stabilization of the negatively charged O1 atom seems to be played by the side-chain amide moiety of Asn170 as compared to the side-chain hydroxyl group of Ser237 in **RTS1**. However, the former seems less efficient than the latter in terms of the stabilization of O1 as reflected in the difference in the associated hydrogen bond distances: 1.81 Å in **HTS1** (Fig. 7) as compared to 1.68 Å in **RTS1** (Fig. 5). The higher activation barrier of the first step of hydrolysis mechanism than that of the recyclisation can thus be attributed to the combined effects of the loss of a hydrogen bond with Ser70 and the weakening of hydrogen-bond stabilization with respect to O1 in **HTS1**.

Further approach of O_W_ to C1 at **HTS1** carries the reaction to the tetrahedral intermediate **HINT** in which a proton from Wat411 is fully transferred to Glu166 with complete formation of the O_W_–C1 bond (Fig. 7). The second step involves dissociation of Ser70–C1 bond and simultaneous proton transfer from the protonated Glu166 to the leaving OG atom of Ser70, leading to formation of **HTS2**. During these changes, the second hydrogen of Wat411 moves from the terminal aminocarbonyl oxygen of avibactam to Glu166 to make a new O–H···O hydrogen bond. We note that only Glu166 plays a substantial role in stabilizing the leaving OG atom of Ser70 in **HTS2** whereas Ser130 and Wat411 donate two hydrogen bonds to stabilize the leaving group in **RTS2** (Fig. 5). The better stabilization of the leaving group (protonated Ser70) in **RTS2** than in **HTS2** rationalise the preference for recyclisation relative to hydrolysis with a large difference (9.2 kcal mol^–1^) in the overall free energies of activation. **HTS2** then decays to form the final minimum energy structure (**HP**), *i.e.* the complex of the hydrolyzed reaction product (Fig. 7). The covalent bond between Ser70 OG and C1 atoms is fully broken in **HP**, leading to restoration of sp^2^ hybridization at the central C1 atom.

### Putative recyclisation of oxacillin mechanism in the OXA-1 active site

**Fig. 8:**
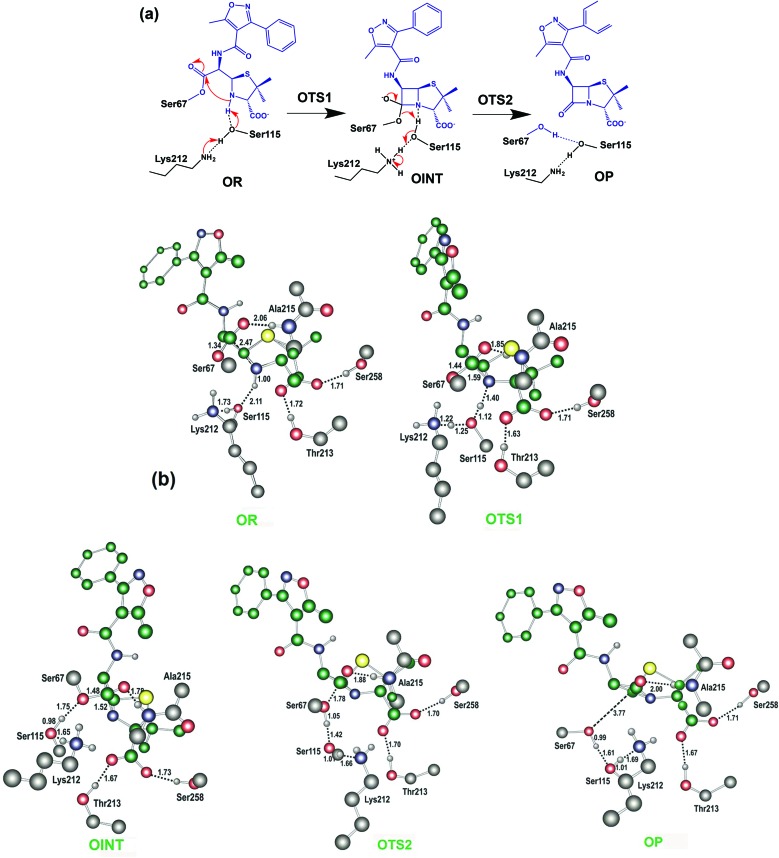
Reaction mechanism of the putative recyclisation of the oxacillin derived acyl-enzyme complex in the OXA-1 active site. (a) **OR**, **OTS1**, **OINT**, **OTS2**, and **OP** denote the reactive complex, first transition state, reaction intermediate, second transition state, and final energy minimum, respectively. (b) Calculated structures of the energy minima and transition states for the enzymatic reaction model for the recyclisation of oxacillin in the active site of OXA-1. Selected interatomic distances are in Å. **OR**, **OTS1**, **OINT**, **OTS2**, and **OP** represent the reactive complex, first transition state, reaction intermediate, second transition state, and final energy minimum, respectively, during the recyclisation reaction.

The results described above imply that the recyclisation of the avibactam derived covalent complex is possible because it involves formation of a five- rather than a four-membered ring. To test this proposal, we investigated the free energy profile for recyclisation of the acyl-enzyme derived from the clinically important β-lactam oxacillin. We worked with the OXA-1 β-lactamase for which a relevant high-resolution crystal structure with oxacillin is available (OXA-1 is a class D β-lactamase) (Fig. 8). As in the case of avibactam, MP2/6-31G*//RHF/6-31G* level of theory was employed to obtain the structures and free energies of the energy minima and transition states (**OR**, **OTS1**, **OINT**, **OTS2**, and **OP** in Fig. 8b) using a crystal structure of OXA-1 complexed ring opened oxacillin (PDB entry: ; 4MLL).^45^

**Fig. 9:**
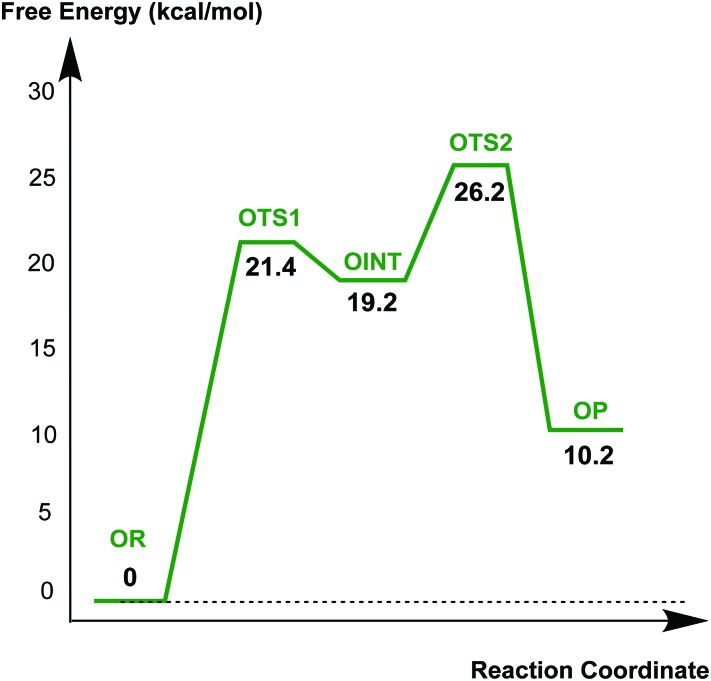
Free energy profile diagram along the enzymatic reaction model for putative recyclisation of the oxacillin derived acyl-enzyme complex in the OXA-1 active site. Free energy is measured from the reactant complex (**OR**) for each stationary-state structure using the MP2/6-31G* level of calculation. **OTS1**, **OINT**, **OTS2**, and **OP** represent the first transition state, reaction intermediate, second transition state, and final energy minimum, respectively, during the recyclisation reaction.

Fig. 9 displays the free energy profile diagram along the reaction coordinate for putative recyclisation of the oxacillin derived acyl-enzyme complex. As for avibactam, the first step involves nucleophilic attack of the β-lactam derived nitrogen in the reactant complex (**OR**) onto the carbonyl of the ring-opened oxacillin to form a tetrahedral intermediate (**OINT**) with a free energy of 19.2 kcal mol^–1^ above **OR**. A calculated barrier of 21.4 kcal mol^–1^ must be overcome in this step to restore the four-membered ring. Cleavage of the C–O bond formed between oxacillin and Ser67 leads to the formation of intact oxacillin (**OP**) that is 10.2 kcal mol^–1^ higher in free energy than **OR**. The activation free energy for this second step is estimated to be 7.0 kcal mol^–1^. The second transition state (**OTS2**) has a higher calculated free energy than the first one (**OTS1**) by 4.9 kcal mol^–1^. The overall activation free energy for the conversion of **OR** to **OP** amounts to 26.2 kcal mol^–1^, which is substantially higher (4.9 kcal mol^–1^) than for recyclisation to form avibactam (Fig. 4). Because the mechanisms of the two catalytic recyclisation reactions are similar, the increased ease of avibactam recyclisation compared to oxacillin is likely predominantly due to the lowering of kinetic barriers to form the five- rather than the four-membered ring.

### Energetic and entropic contributions to the enzymatic reactions

We then estimated the relative importance of enthalpic and entropic contributions to avibactam recyclisation and hydrolysis. Table 1 lists the calculated changes in electronic energy (Δ*E*_elec_), thermal energy including the zero-point vibrational energy (Δ*H*′), and entropic term (–*T*Δ*S*) along the IRCs of the enzymatic reaction models under consideration. The thermodynamic parameters were calculated at 298.15 K and 1 atm using the usual statistical mechanical expressions and RHF/6-31G* vibrational frequencies.^46^ To address the dependences of the free energy profiles of the two enzymatic reaction mechanisms on the basis set and the treatment of electron correlation, we compared the Δ*E*_elec_ values for all the stationary-state structures (as defined in Table 1) calculated at both MP2/6-31G* and wB97X/6-311++G** levels of theory. The latter is a hybrid density functional with long-range correction for non-covalent interactions.^47^

**Table 1:** Relative contributions to the free energy changes of the recyclisation and hydrolysis reactions of avibactam (5-member ring) by CTX-M-15 and the recyclisation of oxacillin (4-member ring) by OXA-1. Numbers in and out of parentheses are the energy values calculated at the MP2/6-31G* and wB97X/6-311++G** levels of theory, respectively. Energies are given in kcal mol^–1^. The results for recyclisation of oxacillin by OXA-1 enzyme are given for comparison

Energy minima and transition states	Δ*E*_elec_	Δ*H*′	–*T*Δ*S*	Δ*G*°
Recyclisation of avibactam by CTX-M-15				
**RR**	0(0)	0	0	0(0)
**RTS1**	18.7(15.5)	–1.9	2.6	19.4(16.2)
**RINT**	12.3(14.2)	–1.5	2.2	12.9(14.9)
**RTS2**	21.3(18.7)	–1.2	1.2	21.3(18.7)
**RP**	8.1(6.6)	–0.2	0.7	8.7(7.1)

Hydrolysis of avibactam by CTX-M-15				
**HR**	0(0)	0	0	0(0)
**HTS1**	26.1(21.1)	–0.9	0.5	25.7(20.7)
**HINT**	18.8(15.9)	–1.1	0.8	18.5(15.6)
**HTS2**	29.7(25.2)	–1.3	2.2	30.5(26.1)
**HP**	10.7(7.4)	–0.1	–0.3	10.3(7.0)

Recyclisation of oxacillin by OXA-1				
**OR**	0(0)	0	0	0(0)
**OTS1**	19.4(16.1)	–4.3	6.3	21.4(18.1)
**OINT**	15.2(11.9)	–0.2	4.2	19.2(15.9)
**OTS2**	27.0(23.3)	–2.7	1.9	26.2(22.5)
**OP**	13.8(9.4)	–1.3	–2.3	10.2(5.8)

The free energy of activation (Δ*G* ^‡^) for recyclisation is calculated to be lower than that of hydrolysis based on the results of both the MP2/6-31G* and wB97X/6-311++G** calculations, *i.e.* 21.3 *versus* 30.5 and 18.7 *versus* 26.1 kcal mol^–1^, respectively. The higher Δ*G* ^‡^ values in MP2 results than in density functional calculations are unsurprising because of tendencies to underestimate and overestimate electron correlation effects, respectively.^48,49^ However, the differences in Δ*E*_elec_ values among the stationary-state structures are similar to those in the corresponding Δ*G* ^‡^ values. Both the MP2/6-31G*//RHF/6-31G* (9.2 kcal mol^–1^) and wB97X/6-311++G**//RHF/6-31G* (7.4 kcal mol^–1^) levels of theory predict a large gap between the activation free energies of the recyclisation and hydrolysis reactions. The consistency between MP2 and DFT results has been observed in calculations on other protein catalysts (*i.e.* theozymes).^50^ Thus, the overall results of MP2 and density functional calculations further support a preference for avibactam recyclisation over hydrolysis.

It is notable that in both recyclisation and hydrolysis of the avibactam complex, the major contribution to the free energy of activation (Δ*G* ^‡^) comes from the activation enthalpy (Δ*H* ^‡^) (Δ*E*_elec_ plus Δ*H*′) rather than the entropy term (–*T*Δ*S* ^‡^). The entropy values amount to only 2.6 and 2.2 kcal mol^–1^ for recyclisation and hydrolysis, respectively, compared to the corresponding Δ*H* ^‡^ values of 20.1 and 28.4 kcal mol^–1^ calculated at the MP2/6-31G* level of theory. The tendency is retained when the method to calculate Δ*E*_elec_ values is changed to the wB97X/6-311++G** level. Thus, recyclisation seems to be preferred over hydrolysis for CTX-M-15 enzyme because the former is more effective than the latter in lowering Δ*H* ^‡^ through the stabilization of the unstable transition states and reaction intermediates by ‘direct’ interactions.

Because the electronic energy term contributes predominantly to free energy changes along the reaction pathways (Table 1), the predicted difference in activation free energies for recyclisation and hydrolysis can likely be rationalized by proton transfers. It is notable in this regard that the required proton transfer reactions are more advanced in **RTS1** and **RTS2** (Fig. 5) than in **HTS1** and **HTS2** (Fig. 7), respectively, towards the formation of the intermediates and products. This proposal is consistent with the knowledge that, in general, an alkoxide is a stronger base than a carboxylate, and an alkyloxonium ion is a stronger acid than a carboxylic acid. It can thus be argued that in the recyclisation mechanism, Lys73-Ser130 dyad may serve not only as the better general base catalyst to activate the *N*-nucleophile in the first step, but also as the better general acid catalyst in the second step than the side chain of Glu166 that plays the similar roles in the hydrolysis mechanism. Hence, the preference for recyclisation over hydrolysis for the deacylation of Ser70 in the CTX-M-15-avibactam complex might in part be associated with better performance of the proton shuttle by Lys73-Ser130 dyad than by Glu166. The effectiveness of Lys-Ser dyad in general acid and general base catalyst has also been appreciated in catalytic reactions of various enzymes including VP4 protease,^51^ penicillin-binding protein 5^52^ and LexA protein^53^ as well as other class A β-lactamases.^54,55^

## Conclusions

Both efficient formation and the stability of the respective covalent complexes are important factors in determining the efficacy of antibiotics/β-lactamase inhibitors working *via* ‘acylating’ type mechanisms, including the β-lactam antibacterials and avibactam. The combined results presented here and previously^37,38^ support the proposal^22^ that the bicyclic core of avibactam enables formation of a highly stable covalent complex with β-lactamases, in a manner comparable to that of β-lactam inhibitors. Our work focused on potential reactions of this complex. Based on MD simulations of CTX-M-15 enzyme in complex with avibactam and quantum chemical calculations on relevant mechanistic reaction models, we examined the possibilities of the competing recyclisation and hydrolysis fates of the β-lactamase-avibactam complex, processes which are relevant to potency and, potentially, resistance. In most of the trajectory snapshots collected from MD simulations, the nitrogen (N_S_) of the hydroxylamine-*O*-sulfonate group of avibactam is closer to the carbonyl carbon (C1) of the carbamoyl moiety than the active site water molecule (Wat411) bound to Glu166. The hydrogen bond between the N_S_ atom of avibactam and Lys73-Ser130 dyad is also observed to be in a stable form during almost the entire course of simulation. The MD simulation results thus support preferential recyclisation over hydrolysis for the avibactam complex. In contrast, analogous calculations imply that the recyclisation of an exemplary β-lactam, oxacillin, is disfavoured, as anticipated based on work with small molecules.^56^ The results of calculations at MP2/6-31G*//RHF/6-31G* and wB97X/6-311++G** levels of theory on the model systems provide further evidence that the avibactam recyclisation mechanism is kinetically favoured over the hydrolysis mechanism with the difference of 9.2 kcal mol^–1^ in the activation free energies, supporting the previous experimental implications.^27,33^ In both deacylation fates, the entropic penalties for the formation of transition states and reaction intermediates were small compared to the variations of electronic energies. Consistent with the difference in activation free energies, recyclisation appears to be more efficient than hydrolysis in terms of stabilizing the negative charges developed on the carbonyl oxygen (O1) of the acyl-enzyme complex and the leaving OG atom of Ser70 of the transition states and the tetrahedral reaction intermediate. The differences in the structural features of the transition states involved in the two deacylation fates indicate that, when compared to the role of Glu166 in the hydrolysis, during recyclisation, the Lys73-Ser130 dyad can serve not only as the better general base to facilitate the nucleophile addition in the first step but also as the better electrophilic catalyst to stabilize the leaving group in the second step. The combination of the results of this study and those of previous work^27,33,34,38^ implies that the avibactam template is not only optimized for binding to give a covalent complex, but that recyclisation to reform active avibactam is favoured over hydrolysis to give an inactive product. In this sense avibactam, and other reversibly and covalently binding inhibitors, may thus have an advantage over (at least bicylic) β-lactam inhibitors which recyclisation appears not be an option as shown by our calculations on the acyl-enzyme complex formed from oxacillin.

By any reasonable standards the β-lactams are exemplars of the therapeutic power of covalent enzyme-inhibition *via* acylation of a nucleophilic residue. The special properties of β-lactams have been proposed in part to arise from their ability to acylate a nucleophilic active site residue which is unusually stable with respect to both recyclisation and, when appropriately functionalised, to hydrolysis.^18^ In contrast, since the formation of larger lactam ring sizes can be chemically easier than β-lactams for stereoelectronic reasons,^56^ they can undergo more facile recyclisation, a property that might normally be considered disfavourable in terms of potency.^16,18^ However, when considering resistance, reversible reaction of a covalently reacting inhibitor may be a useful property since recyclisation to reform an active inhibitor is referable to hydrolysis yielding an inactive one.^16,17^ The expanded ring sizes may be favourable in this regard, and the inability of β-lactams to undergo recyclisation could be regarded an Achilles heel compared to analogous reversibly reacting inhibitors. With the expanding clinical use of the breakthrough non-β-lactam β-lactamase inhibitor avibactam and likely inspired successors, time will tell.

## Experimental methods

### MD simulations

MD simulations of CTX-M-15 enzyme in complex with avibactam were carried out using the AMBER program (version 12) and the standard force field parameters.^57^ To obtain the potential parameters for the avibactam molecule which were unavailable in the force field database, we followed the procedure of Fox and Kollman^58^ to be consistent with the standard AMBER force field. We chose to work with CTX-M-15 because of its clinical importance and because of the availability of high-resolution crystal structures. The starting coordinates for MD simulations were derived from a crystal structure of CTX-M-15 complexed with avibactam (PDB entry: ; 4HBU).^34^ To obtain the all-atom model including hydrogens for CTX-M-15, the protonation states of ionizable residues were assigned by inspecting their hydrogen bonding patterns in the crystal structure. For example, the side chains of Asp and Glu residues were assigned as neutral if either of their carboxylate oxygens was directed toward a hydrogen-bond accepting group within the distance of 3.5 Å (generally accepted distance limit for hydrogen bonds of moderate strength).^40^ Similarly, the side chains of lysine residues were assumed to be protonated unless the NZ atom was in proximity to a hydrogen-bond donating group. The same procedure was also applied for determining the protonation states of His residues.

After the addition of 3 chloride ions to neutralize total charge, the all-atom model for the CTX-M-15-avibactam complex was placed in a rectangular box of dimension 60.9 × 75.4 × 71.2 Å containing 8475 TIP3P^59^ water molecules. After 2000 minimization cycles to remove poor steric contacts, the system was equilibrated beginning with 20 ps equilibration dynamics of solvent molecules at 300 K. The next step involved the equilibration of the solutes with a fixed configuration of the solvent molecules consecutively at 10, 50, 100, 150, 200, 250, and 300 K for 10 ps at each temperature. Equilibration dynamics of the entire system were then performed at 300 K for 100 ps. Following equilibration, 10.8 ns production dynamics simulations were carried out with periodic boundary conditions in the NPT ensemble. The temperature and pressure were kept at 300 K and 1 atm using Berendsen temperature coupling^60^ and isotropic molecule-based scaling, respectively. The SHAKE algorithm,^61^ with a tolerance of 10^–6^ Å, was applied to fix all bond lengths involving hydrogen atoms. We used a time step of 2.0 fs and a nonbond-interaction cut-off radius of 12 Å; the trajectory was sampled every 0.4 ps (200-step intervals) for analysis.

### Quantum mechanical calculations

In the simplified model systems for quantum studies on the relative energetics of recyclisation *versus* hydrolysis of the avibactam derived acyl-enzyme propionate, acetamide, methanol, and butylamine were used to mimic the side chains of Glu, Asn, Ser, and Lys residues in the active site of CTX-M-15, respectively. Two structurally observed water molecules, Wat411 and Wat490, were included in models because they interact with both avibactam and active-site residues in X-ray analyses.^32^

All structures corresponding to energy minima and transition states on the model enzymatic reaction pathways were optimized at the RHF/6-31G* level using the GAMESS program.^62^ Geometry optimizations were performed using analytically determined gradients and quasi-Newton–Raphson optimization algorithms.^63^ The nature of each stationary-structure encountered on reaction pathways was determined by the number of imaginary frequencies obtained by diagonalization of the analytical Hessian matrix. Each transition state was identified to have a single negative eigenvalue; the corresponding imaginary vibrational frequency was related to the motion that would connect the expected starting and final minima. The intrinsic reaction coordinate (IRC) connecting a transition state to its neighboring energy minima was determined using the Gonzalez–Schlegel second-order (GS2) method^64^ at the same level as used in geometry optimizations.

To obtain the better prediction for the energetics, post-HF level calculations including the effect of electron correlation were conducted at the optimized stationary-state structures. These single point calculations were carried out using the 6-31G* basis set using Møller–Plesset second-order perturbation theory (MP2).^65^ We also carried out the single point calculations on transition intermediates/stationary structures in the reactions using density functional theory at the wB97X/6-311++G** level.^48^ The electronic energies computed in this way were used to calculate the relative free energies (Δ*G*) given by the following equation.

1 Δ*G* = Δ*E*_elec_ + Δ*H*′ – *T*Δ*S*

Δ*H*′ denotes the enthalpy change due to thermal motions of the nuclei including the zero-point vibrational energies, and Δ*S* is the entropy change. Electronic energies (*E*_elec_) were evaluated at MP2/6-31G* and wB97X/6-311++G** levels of theory and RHF/6-31G* vibrational frequencies were used to calculate Δ*H*′ and Δ*S*; thermal contributions were evaluated at 298 K.
