# Supplementary material for: Investigations on recyclisation and hydrolysis in avibactam mediated serine β-lactamase inhibition
Source: Org Biomol Chem. 2016 Apr 13;14(17):4116–28. doi: 10.1039/c6ob00353b (PMC4847122; doi:10.1039/c6ob00353b)
Supplement: Supplementary file 1 [file OB-014-C6OB00353B-s001.pdf]

Electronic Supplementary Material (ESI) for Organic & Biomolecular Chemistry.
This journal is © The Royal Society of Chemistry 2016

Supplementary Information

Investigations on Recyclisation and Hydrolysis in Avibactam Mediated Serine β-Lactamase Inhibition

Hwanho Choi,[†, ‡] Robert S. Paton,[†] Hwangseo Park,[*, ‡] and Christopher J. Schofield[*, †]

[†] Department of Bioscience and Biotechnology and Institute of Bioscience, Sejong University, 209 Neungdong-ro, Kwangjin-gu, Seoul 143-747, Korea

[‡] Chemical Research Laboratory, University of Oxford, Mansfield Road, Oxford OX1 3TA, United Kingdom

Telephone: +82 2 3408 3766; +44 (0) 1865 275 625

Fax: +82 2 3408 3334; +44 (0) 1865 285 022

E-mail: hspark@sejong.ac.kr; christopher.schofield@chem.ox.ac.uk

1. MD SIMULATIONS OF THE β-LACTAMASE-AVIBACTAM COMPLEX 2

1.1 COMPARISON OF PROTEIN STRUCTURE STABILITY BETWEEN PROTONATED AND DEPROTONATED FORMS OF GLU166..2

2. MP2/6-31G* AND WB97X/6-311++G CALCULATIONS 5**

2.1 STATIONARY POINTS: 5

2.2 CARTESIAN COORDINATES (RHF/6-31G* LEVEL) : 6

3. SUPPLEMENTARY REFERENCES 21

1. MD SIMULATIONS OF THE β-LACTAMASE AVIBACTAM COMPLEX.

1.1. Comparison of protein structure stability between protonated and deprotonated forms of Glu166

To examine the maintenance of protein structure under the simulation conditions, we calculated the root-mean-square deviations of backbone heavy atoms from the starting structure of production dynamics ($RMSD_{init}$) during the entire simulation time for the two CTX-M-15 avibactam complexes differing in the Glu166 protonation state (Fig. S1). The $RMSD_{init}$ values remained within 1.5 and 1.2 Å for both the neutral and deprotonated Glu166 structures (Fig. S2), respectively, with the corresponding average values of 0.95 and 0.90 Å; these results indicate that the modeled protein is conformationally stable during the simulations independent of the Glu166 protonation state. These results also suggest that the CTX-M-15 avibactam complex favors the deprotonated form of Glu166.

The deprotonated Glu166 plays an important role in the catalytic action of CTX-M-15 (and, at least most other, serine β-lactamases); therefore, knowledge of its protonation state is important in mechanistic investigations. Despite knowledge from crystal structures of CTX-M-15,[1] it is difficult to assign the protonation state of Glu166 in an unambiguous manner, in part due to the presence of a 'structural' water molecule (Wat411) in its vicinity. In contrast to the assigned preference of the ionized form of Glu-166 in the apo CTX-M-15 structure,[1] the neutral form of Glu166 was proposed to be favored in the crystal structure of CTX-M-15 avibactam complex.[1] However, the role of deprotonated Glu166 in the deacylation mechanism of Class A enzymes cannot be generalized because of the possibility that Glu166 plays a role as a general base to activate a nearby water (Wat411, (Fig. S1) in order to hydrolyze the avibactam acyl-enzyme; such hydrolysis occurs, albeit slowly, with another serine β-lactamase KPC-2 whose active-site residues occupy very similar positions to those of CTX-M-15.[2,3]

On the basis of the crystallographically observed interatomic distances between Wat411, Glu166, Asn170, and Ser70 observed in the structure of CTX-M-15-avibactum complex,[1] two possibilities for the hydrogen-bonding patterns may be taken into account for the Glu-166 protonation state (Fig. S1). The first involves the deprotonated form of Glu166, with Wat411 playing the role of a hydrogen bond donor with respect to Glu166 and Ser70 (Fig. S1a). Alternatively, Glu166 may exist in the neutral form in which Wat411 receives and donates the hydrogen bonds from Glu166 and to Asn170 and Ser70 (Fig. S1b), respectively. We investigated the preferred protonation state of Glu166 by comparing stabilities of the hydrogen bond between Glu166 and Wat411 based on MD simulations the CTX-M-15 avibactam complex.

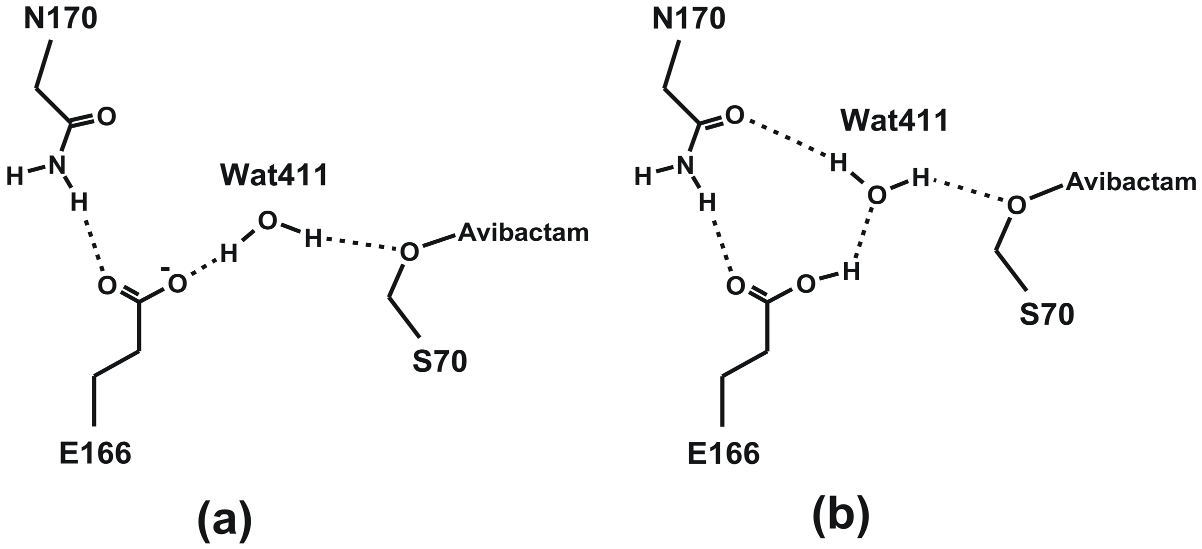

Supplementary Fig. 1 Possible protonation states of Glu166. Crystallographic analysis of the CTX-M-15-avibactam complex represented by (a) ionized and (b) neutral forms. Dotted lines indicate potential hydrogen bonds. The structures were prepared by extracting the atomic coordinates from a CTX-M-15-avibactam complex X-ray structure.

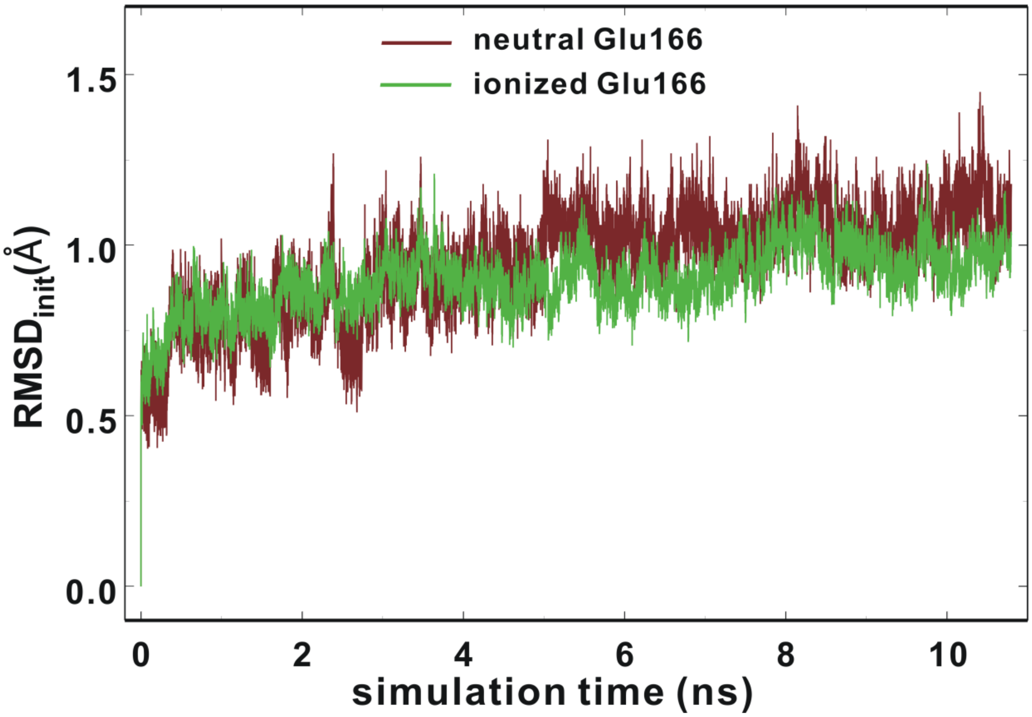

Supplementary Fig. 2 Comparative view of the time evolutions between protonated and deprotonated forms of Glu166. $RMSD_{init}$ values for protein backbone heavy atoms in CTX-M-15 avibactam complexes with protonated (brown) and deprotonated (green) forms of Glu166 as determined by molecular dynamic simulation of AMBER force field (ver. 12) (see Fig. S1).

2. MP2/6-31G* AND WB97X/6-311++G** CALCULATIONS

2.1 Stationary points:

All stationary points were fully optimized at the RHF/6-31G* level of theory using the GAMESS program.[4] Geometry optimizations were performed using analytically determined gradients and quasi-Newton-Raphson optimization algorithms.

Each assigned transition state structure was identified to have a single negative eigenvalue and the corresponding imaginary vibrational frequency was related to the motion that connects the expected starting and final minima. The intrinsic reaction coordinate (IRC) connecting a transition state to a neighboring energy minima was determined using the Gonzalez-Schlegel second-order (GS2) method[5] at the same level of theory as used in the geometry optimizations. Post-Hartree-Fock level calculations including the effects of electron correlation were conducted for the optimized stationary-state structures. These single point calculations were carried out with the 6-31G* basis set using Møller−Plesset second-order perturbation theory (MP2).[6] Considering the possibility of underestimating the electron correlation effects, we also carried out single point calculations based on the density functional theory at wB97X/6-311++G** level. The figures in the main text (Fig. 5 and Fig. 7) for the calculated structures of recyclisation of avibactam in the active site of CTX-M-15 and hydrolytic cleavage of Ser70-avibactam in the active site of CTX-M-15 are based on the Cartesian coordinates as defined in Section 2.2.

2.2 Cartesian coordinates:

RHF/6-31G* level of calculation for Geometric optimization

Reversible Avibactam stationary points:

Reversible Reactant

RHF/6-31G*

C	-0.9920	-1.4660	0.5040
N	-1.1640	-1.5000	-0.8260
C	-2.4580	-1.9120	-1.3460
C	-3.1110	-0.8090	-2.2280
C	-2.1450	0.0010	-3.1000
C	-0.9410	0.4430	-2.2730
C	-0.2500	-0.8200	-1.7520
O	-1.7830	-1.9080	1.3340
C	-2.3810	-3.2460	-2.0670
N	-3.6020	-3.7580	-2.3140
O	-1.3370	-3.8190	-2.3710
N	-1.4010	1.1840	-1.0710
O	-1.9340	2.4880	-1.5470
S	-3.2830	2.9290	-0.8180
O	-4.3370	2.0370	-1.2960
O	-3.4350	4.2920	-1.2930
O	-3.0620	2.8110	0.6050
H	-3.1030	-2.0670	-0.4960
H	-4.4380	-3.3200	-1.9470
H	-3.6570	-4.6470	-2.7610
H	-3.8800	-1.2630	-2.8350
H	-3.5800	-0.1130	-1.5680
H	-1.7830	-0.5780	-3.9450
H	-2.6760	0.8660	-3.4760
H	-0.2350	1.0120	-2.8600
H	0.6670	-0.5870	-1.2550
H	-0.0380	-1.4810	-2.5770
H	-0.6300	1.4590	-0.4640
O	0.1640	-0.8660	0.8560
C	0.3970	-0.4280	2.2300
H	0.9640	-1.1880	2.7680
H	-0.5550	-0.2570	2.7080
H	0.9260	0.5000	2.1240
H	2.1950	-1.4000	0.2170
O	2.7370	-2.1470	-0.0600
H	3.7170	-1.9440	-0.0170
O	0.7420	2.3310	0.5790
C	0.1030	3.6210	0.5640
H	0.5550	4.3390	1.2480
H	1.5440	2.2740	-0.0250
H	-0.9130	3.4590	0.8730
H	0.0820	4.0420	-0.4310
H	-2.2910	5.5580	-0.9030
O	-1.5290	6.1710	-0.8050
H	-0.9330	6.0000	-1.5450
H	-5.4910	-0.0900	2.1080
C	-4.6660	0.0520	1.4100
O	-5.2300	-0.0260	0.0730
H	-4.9940	0.7790	-0.4580
H	-4.2120	1.0230	1.5410
H	-3.9080	-0.7060	1.5360
H	2.8690	-1.8350	-2.0300
N	3.4950	-1.5280	-2.7530
C	3.2470	-0.4160	-3.4280
C	4.3970	0.0170	-4.3270
O	2.1940	0.2410	-3.3630
H	4.4510	-1.8580	-2.6470
H	4.3440	-0.5340	-5.2610
H	5.3410	-0.2080	-3.8490
H	4.3070	1.0730	-4.5390
O	5.2830	-1.5380	-0.0130

C 6.2250 -1.7030 -0.8420
C 7.6420 -1.5620 -0.2620
C 8.7280 -1.8760 -1.2930
O 6.1230 -1.9570 -2.0650
H 7.7040 -2.2190 0.5980
H 7.7380 -0.5480 0.1130
H 9.7220 -1.7530 -0.8720
H 8.6220 -1.2230 -2.1490
H 8.6210 -2.8920 -1.6500
H 1.6000 -3.5670 -0.1190
N 0.7450 -4.1070 -0.1950
C 0.3220 -4.8550 0.8200
C -1.0870 -5.4010 0.6550
O 0.9660 -5.0590 1.8560
H 0.1650 -3.9630 -1.0010
H -1.2940 -5.6580 -0.3740
H -1.7730 -4.6150 0.9590
H -1.2180 -6.2550 1.3030
N 2.8420 2.2190 -1.0950
C 4.0640 1.6380 -0.4800
C 4.1720 2.0400 0.9990
C 5.3980 1.4020 1.6790
C 5.3800 1.6300 3.2050
H 2.5580 1.7310 -1.9310
H 2.9170 3.2130 -1.2320
H 4.9660 1.9490 -1.0070
H 4.0100 0.5620 -0.5510
H 3.2670 1.7240 1.5050
H 4.2210 3.1220 1.0740
H 6.3050 1.8340 1.2590
H 5.4140 0.3430 1.4530
H 6.2410 1.1670 3.6780
H 4.4810 1.2080 3.6370
H 5.3990 2.6920 3.4340
H -5.4970 -1.4260 -0.7080
O -5.7820 -2.2460 -1.1960
H -6.4360 -1.9800 -1.8560

Revesible Transition State 1

RHF/6-31G*

C 1.1130 1.0790 0.2860
N 1.2040 1.7170 -0.9450
C 2.4810 2.3860 -1.1750
C 3.6030 1.3820 -1.5520
C 3.1360 0.2220 -2.4470
C 1.7740 -0.3270 -2.0030
C 0.7800 0.8500 -2.0580
O 1.8360 1.3480 1.2660
C 2.3300 3.5360 -2.1550
N 3.4340 4.2990 -2.2080
O 1.3100 3.7830 -2.8050
N 1.6750 -0.7320 -0.5710
O 3.0540 -1.1040 -0.0860
S 3.4710 -2.6220 -0.0920
O 4.8350 -2.5780 0.4090
O 3.3850 -3.0430 -1.4920
O 2.5540 -3.3460 0.7650
H 2.7710 2.8450 -0.2470
H 4.2040 4.1650 -1.5520
H 3.4200 5.0960 -2.8070
H 4.4320 1.9080 -2.0020
H 3.9430 0.9480 -0.6320
H 3.0340 0.5530 -3.4790
H 3.8670 -0.5730 -2.4190
H 1.4700 -1.1250 -2.6690
H -0.2350 0.5320 -1.9100
H 0.8480 1.4080 -2.9750
H 0.2790 -1.7090 -0.4360
O -0.1780 0.6510 0.5060

C	-0.5020	0.0260	1.7780
H	-1.1680	0.7000	2.3170
H	0.4120	-0.1330	2.3250
H	-0.9610	-0.9170	1.5490
H	-1.5950	0.6260	-0.4720
O	-2.4530	0.7100	-0.9240
H	-3.1720	0.8790	-0.2600
O	-0.5210	-2.2970	-0.6060
C	-0.0330	-3.5520	-1.1280
H	0.0050	-4.3180	-0.3540
H	-2.2390	-2.0450	-0.8210
H	0.9650	-3.4200	-1.5050
H	-0.6780	-3.8640	-1.9410
H	2.8560	-4.4370	-2.3260
O	2.3680	-5.0970	-2.8730
H	1.9200	-4.5980	-3.5690
H	5.0220	0.6200	2.0860
C	4.9620	1.5730	2.6120
O	4.1490	2.5120	1.8750
H	3.2600	2.1250	1.6890
H	4.5410	1.4190	3.6010
H	5.9420	2.0100	2.7140
H	-3.7660	0.4690	-2.2410
N	-4.7620	0.4810	-2.4040
C	-5.3910	-0.6230	-2.7640
C	-6.9040	-0.4920	-2.7900
O	-4.8280	-1.6980	-3.0500
H	-5.2670	1.1950	-1.8850
H	-7.2040	0.0430	-3.6850
H	-7.2260	0.0800	-1.9280
H	-7.3500	-1.4750	-2.7960
O	-4.3940	1.0820	0.8060
C	-5.2520	1.9940	0.5420
C	-5.2830	3.1640	1.5200
C	-6.4670	4.1020	1.2770

O	-6.0020	2.0250	-0.4580
H	-4.3360	3.6790	1.3850
H	-5.2860	2.7600	2.5260
H	-6.4290	4.9620	1.9390
H	-7.4060	3.5820	1.4360
H	-6.4540	4.4430	0.2510
H	-1.8870	2.5760	-1.2030
N	-1.4070	3.4560	-1.3020
C	-1.4620	4.3600	-0.3290
C	-0.6220	5.6060	-0.5730
O	-2.1500	4.2590	0.6950
H	-0.6600	3.4930	-1.9720
H	0.1400	5.4330	-1.3160
H	-0.1800	5.9160	0.3640
H	-1.2790	6.4010	-0.9120
N	-3.1780	-2.4600	-0.9670
C	-4.2020	-2.0200	0.0640
C	-3.8830	-2.5180	1.4770
C	-4.9850	-2.0180	2.4350
C	-4.7410	-2.4780	3.8850
H	-3.5600	-2.2100	-1.8910
H	-3.0180	-3.4660	-0.8970
H	-5.1470	-2.4250	-0.2710
H	-4.2470	-0.9460	0.0500
H	-2.9310	-2.1250	1.8100
H	-3.8240	-3.6010	1.4900
H	-5.9500	-2.3860	2.0940
H	-5.0080	-0.9360	2.3750
H	-5.5290	-2.1190	4.5410
H	-3.7940	-2.0960	4.2410
H	-4.7220	-3.5630	3.9420
H	4.8360	3.4750	0.6160
O	5.3010	3.7620	-0.2050
H	5.9870	3.0900	-0.3350

Reversible Intermediate

RHF/6-31G*

C	1.2420	-0.5140	-0.1330
N	0.9190	-1.4180	0.9910
C	2.0410	-2.3330	1.2400
C	3.3430	-1.6050	1.7040
C	3.0890	-0.3510	2.5610
C	1.8580	0.4200	2.0760
C	0.6520	-0.5270	2.1410
O	1.9210	-1.0010	-1.1220
C	1.6800	-3.4610	2.1870
N	2.5500	-4.4810	2.0990
O	0.7150	-3.4740	2.9590
N	1.8610	0.7130	0.6130
O	3.2250	0.9350	0.1370
S	3.5760	2.4920	-0.0290
O	4.9670	2.4380	-0.4150
O	3.3590	3.0480	1.3030
O	2.6870	3.0410	-1.0250
H	2.2580	-2.7940	0.2940
H	3.3150	-4.4780	1.4220
H	2.4040	-5.2700	2.6910
H	3.9860	-2.3020	2.2230
H	3.8490	-1.3030	0.8080
H	2.9110	-0.6220	3.5980
H	3.9610	0.2890	2.5260
H	1.7340	1.3390	2.6240
H	-0.2820	-0.0120	1.9870
H	0.6070	-1.1010	3.0490
H	-0.4490	1.5340	0.1890
O	-0.0690	0.0310	-0.5510
C	-0.2900	0.2090	-1.9770
H	-0.8980	-0.5990	-2.3850
H	0.6680	0.2350	-2.4650

H	-0.7940	1.1540	-2.1000
H	-1.6050	-0.3660	0.3250
O	-2.4870	-0.4440	0.7340
H	-3.1800	-0.6120	0.0440
O	-0.9400	2.3010	0.5600
C	-0.0830	3.4160	0.8840
H	-0.0640	4.1430	0.0720
H	-2.5760	2.1920	0.9290
H	0.9200	3.0740	1.0520
H	-0.4680	3.8760	1.7850
H	2.7640	4.4730	2.0580
O	2.2360	5.1540	2.5330
H	1.6870	4.6810	3.1730
H	5.7640	-0.7290	-1.2540
C	5.1700	-1.2770	-1.9850
O	4.1780	-2.1010	-1.3740
H	3.2880	-1.6230	-1.2770
H	4.6990	-0.5460	-2.6320
H	5.8170	-1.9060	-2.5860
H	-3.8610	-0.3010	2.0520
N	-4.8540	-0.4110	2.1890
C	-5.6010	0.5780	2.6390
C	-7.0900	0.2760	2.6420
O	-5.1640	1.6850	3.0210
H	-5.2820	-1.1410	1.6200
H	-7.3310	-0.3300	3.5100
H	-7.3420	-0.2860	1.7520
H	-7.6420	1.2030	2.6920
O	-4.3940	-0.8810	-1.0090
C	-5.2250	-1.8310	-0.8040
C	-5.2390	-2.9360	-1.8540
C	-6.4570	-3.8530	-1.7240
O	-5.9670	-1.9490	0.1990
H	-4.3170	-3.4870	-1.6910
H	-5.1760	-2.4740	-2.8310

H	-6.4000	-4.6840	-2.4220
H	-7.3740	-3.3050	-1.9150
H	-6.5100	-4.2380	-0.7160
H	-2.1080	-2.3150	1.1020
N	-1.7230	-3.2230	1.3090
C	-1.7370	-4.1850	0.3940
C	-0.9290	-5.4230	0.7600
O	-2.3160	-4.1220	-0.6980
H	-1.0480	-3.2550	2.0510
H	-0.8780	-5.5620	1.8290
H	0.0820	-5.3050	0.3850
H	-1.3740	-6.2830	0.2790
N	-3.5150	2.6270	1.0400
C	-4.4260	2.2220	-0.1050
C	-3.8680	2.6790	-1.4550
C	-4.7350	2.1090	-2.5950
C	-4.1780	2.4830	-3.9820
H	-3.9680	2.3490	1.9260
H	-3.3180	3.6240	1.0060
H	-5.3930	2.6660	0.0900
H	-4.5050	1.1490	-0.0930
H	-2.8540	2.3120	-1.5500
H	-3.8350	3.7610	-1.5020
H	-5.7500	2.4890	-2.4930
H	-4.7700	1.0320	-2.4780
H	-4.8410	2.1320	-4.7670
H	-3.2080	2.0300	-4.1360
H	-4.0820	3.5610	-4.0800
H	4.3410	-3.5260	-0.4930
O	4.5540	-4.2150	0.1850
H	5.4640	-4.0130	0.4420

Reversible Transition State 2

RHF/6-31G*

C	-1.5740	0.4450	-0.2010
N	-1.0760	1.3560	0.7810
C	-2.1760	2.3210	1.0390
C	-3.4680	1.6010	1.5450
C	-3.1930	0.3800	2.4500
C	-1.9750	-0.4220	1.9730
C	-0.7540	0.5100	1.9600
O	-1.9810	0.7710	-1.3070
C	-1.8000	3.4570	1.9670
N	-2.6160	4.5120	1.7930
O	-0.8950	3.4360	2.8010
N	-2.0300	-0.7210	0.5100
O	-3.3590	-1.0520	0.0490
S	-3.6680	-2.6470	-0.0410
O	-5.0730	-2.6100	-0.3640
O	-3.3730	-3.1210	1.3020
O	-2.8120	-3.1900	-1.0600
H	-2.4050	2.7700	0.0890
H	-3.3580	4.5010	1.0940
H	-2.4890	5.3060	2.3820
H	-4.1060	2.3140	2.0470
H	-3.9950	1.2680	0.6680
H	-2.9900	0.6980	3.4680
H	-4.0700	-0.2550	2.4650
H	-1.8500	-1.3380	2.5170
H	0.1610	-0.0210	1.7680
H	-0.6670	1.1280	2.8320
H	0.7030	-1.2690	-0.1100
O	0.3570	-0.3380	-0.6700
C	0.5820	-0.4680	-2.0800
H	1.1960	0.3430	-2.4720
H	-0.3730	-0.4520	-2.5890
H	1.0700	-1.4090	-2.2950
H	1.5620	0.5520	0.1940
O	2.3390	0.9050	0.6800

H	3.1340	0.9030	0.0920
O	1.1900	-2.2500	0.5130
C	0.2900	-3.3110	0.8110
H	0.3350	-4.1090	0.0700
H	2.4610	-2.3330	1.0280
H	-0.7250	-2.9470	0.8170
H	0.5080	-3.7310	1.7920
H	-2.6340	-4.5280	2.0480
O	-2.0600	-5.1830	2.5000
H	-1.4040	-4.6780	2.9970
H	-5.9150	0.5450	-1.3880
C	-5.4010	1.0800	-2.1870
O	-4.3520	1.9300	-1.6920
H	-3.4870	1.4640	-1.6580
H	-4.9920	0.3440	-2.8680
H	-6.0990	1.7060	-2.7260
H	3.5760	0.6470	2.1560
N	4.5470	0.7600	2.4030
C	5.2190	-0.1740	3.0500
C	6.7050	0.1280	3.1860
O	4.7290	-1.2210	3.5150
H	5.0550	1.4270	1.8270
H	6.8560	0.8180	4.0110
H	7.0600	0.5980	2.2780
H	7.2380	-0.7890	3.3900
O	4.5090	0.9580	-0.8340
C	5.2820	1.9480	-0.6090
C	5.3240	3.0160	-1.7000
C	6.4910	3.9900	-1.5210
O	5.9390	2.1430	0.4400
H	4.3720	3.5370	-1.6340
H	5.3540	2.5160	-2.6600
H	6.4490	4.7950	-2.2490
H	7.4410	3.4760	-1.6280
H	6.4580	4.4090	-0.5250
H	1.8940	2.5900	0.8770
N	1.5350	3.5330	1.0320
C	1.6240	4.4650	0.0950
C	0.9160	5.7750	0.4320
O	2.2060	4.3320	-0.9890
H	0.9210	3.6410	1.8130
H	1.2480	6.1440	1.3950
H	-0.1550	5.6100	0.4810
H	1.1360	6.4980	-0.3390
N	3.5160	-2.5820	1.3670
C	4.5020	-2.1260	0.3180
C	4.1520	-2.7060	-1.0570
C	5.0950	-2.1290	-2.1310
C	4.6900	-2.5730	-3.5500
H	3.7620	-2.1570	2.2660
H	3.4950	-3.5930	1.4290
H	5.4930	-2.4340	0.6270
H	4.4640	-1.0500	0.2700
H	3.1260	-2.4440	-1.2800
H	4.2180	-3.7890	-1.0340
H	6.1120	-2.4540	-1.9210
H	5.0690	-1.0480	-2.0540
H	5.4160	-2.2300	-4.2820
H	3.7270	-2.1570	-3.8180
H	4.6340	-3.6570	-3.6140
H	-4.4350	3.4360	-0.8170
O	-4.6240	4.1110	-0.1280
H	-5.5160	3.8920	0.1750

Reversible Products

RHF/6-31G*

C	1.9430	-0.5460	-0.3040
N	1.4010	-1.6510	0.4130
C	2.5530	-2.5770	0.6130

C	3.7160	-1.8630	1.3770
C	3.2510	-0.8450	2.4480
C	2.0080	-0.0610	1.9980
C	0.8970	-1.0770	1.6940
O	2.3520	-0.5670	-1.4400
C	2.1910	-3.8950	1.2690
N	3.1390	-4.8160	1.0390
O	1.1920	-4.1070	1.9560
N	2.1720	0.4950	0.6250
O	3.4840	1.0390	0.3870
S	3.6040	2.6680	0.4170
O	5.0390	2.8000	0.3660
O	3.0040	3.0250	1.6890
O	2.8990	3.1550	-0.7360
H	2.9120	-2.8230	-0.3710
H	3.9360	-4.6160	0.4380
H	3.0190	-5.7230	1.4340
H	4.3660	-2.6040	1.8170
H	4.2950	-1.3410	0.6340
H	2.9910	-1.3550	3.3690
H	4.0660	-0.1660	2.6580
H	1.7430	0.7310	2.6740
H	-0.0610	-0.6280	1.5170
H	0.8210	-1.8540	2.4300
H	-0.8040	1.1660	-0.4660
O	-0.5670	0.2410	-0.7960
C	-0.7980	0.1300	-2.2150
H	-1.5010	-0.6750	-2.4280
H	0.1430	-0.0920	-2.6960
H	-1.1950	1.0560	-2.6080
H	-1.7160	-0.5920	0.2170
O	-2.3930	-1.0040	0.7880
H	-3.2680	-0.9670	0.3200
O	-1.3100	2.5310	-0.0110
C	-0.3620	3.5030	0.4510

H	-0.2310	4.2900	-0.2920
H	-2.2400	2.6350	0.4230
H	0.5860	3.0170	0.6020
H	-0.6900	3.9370	1.3890
H	2.1990	4.4960	2.2740
O	1.6160	5.2230	2.5770
H	0.8580	4.8150	3.0160
H	6.2800	-0.0580	-1.1000
C	5.8980	-0.4820	-2.0290
O	4.8890	-1.4850	-1.8000
H	3.9890	-1.1030	-1.8170
H	5.4890	0.3250	-2.6220
H	6.7000	-0.9540	-2.5810
H	-3.5720	-0.3520	2.3620
N	-4.5420	-0.3740	2.6250
C	-5.1290	0.6640	3.2060
C	-6.6340	0.4870	3.3650
O	-4.5530	1.6880	3.6000
H	-5.1100	-1.0520	2.1240
H	-6.8330	-0.0830	4.2680
H	-7.0310	-0.0600	2.5200
H	-7.0970	1.4590	3.4570
O	-4.6760	-0.9730	-0.5470
C	-5.5440	-1.8410	-0.2030
C	-5.8170	-2.9470	-1.2220
C	-7.0740	-3.7530	-0.8870
O	-6.1270	-1.8950	0.9050
H	-4.9340	-3.5800	-1.2040
H	-5.8730	-2.4930	-2.2040
H	-7.1980	-4.5920	-1.5660
H	-7.9590	-3.1270	-0.9460
H	-7.0030	-4.1220	0.1270
H	-1.9630	-2.7240	0.8580
N	-1.5860	-3.6680	0.9530
C	-1.9860	-4.6730	0.1830

C -1.3320 -6.0140 0.5080

O -2.8250 -4.5900 -0.7220

H -0.7800 -3.7790 1.5340

H -0.3540 -5.8750 0.9430

H -1.2740 -6.6010 -0.3970

H -1.9600 -6.5420 1.2190

N -3.6630 2.9500 1.0160

C -4.6810 2.3410 0.1130

C -4.3760 2.6620 -1.3580

C -5.3530 1.9210 -2.2910

C -4.9820 2.0920 -3.7780

H -3.7950 2.6450 1.9730

H -3.6450 3.9550 0.9470

H -5.6840 2.6870 0.3610

H -4.6510 1.2700 0.2430

H -3.3600 2.3550 -1.5690

H -4.4300 3.7360 -1.5250

H -6.3610 2.2940 -2.1200

H -5.3380 0.8720 -2.0230

H -5.7410 1.6490 -4.4180

H -4.0410 1.6020 -3.9960

H -4.8970 3.1440 -4.0360

H 5.0450 -3.1270 -1.1730

O 5.2430 -3.8850 -0.5810

H 6.0850 -3.6470 -0.1680

Hydrolysis Avibactam stationary points:

Hydrolysis Reactant

RHF/6-31G*

C -0.9920 -1.4660 0.5040

N -1.1640 -1.5000 -0.8260

C -2.4580 -1.9120 -1.3460

C -3.1110 -0.8090 -2.2280

C -2.1450 0.0010 -3.1000

C -0.9410 0.4430 -2.2730

C -0.2500 -0.8200 -1.7520

O -1.7830 -1.9080 1.3340

C -2.3810 -3.2460 -2.0670

N -3.6020 -3.7580 -2.3140

O -1.3370 -3.8190 -2.3710

N -1.4010 1.1840 -1.0710

O -1.9340 2.4880 -1.5470

S -3.2830 2.9290 -0.8180

O -4.3370 2.0370 -1.2960

O -3.4350 4.2920 -1.2930

O -3.0620 2.8110 0.6050

H -3.1030 -2.0670 -0.4960

H -4.4380 -3.3200 -1.9470

H -3.6570 -4.6470 -2.7610

H -3.8800 -1.2630 -2.8350

H -3.5800 -0.1130 -1.5680

H -1.7830 -0.5780 -3.9450

H -2.6760 0.8660 -3.4760

H -0.2350 1.0120 -2.8600

H 0.6670 -0.5870 -1.2550

H -0.0380 -1.4810 -2.5770

H -0.6300 1.4590 -0.4640

O 0.1640 -0.8660 0.8560

C 0.3970 -0.4280 2.2300

H 0.9640 -1.1879 2.7678

H -0.5550 -0.2570 2.7080

H 0.9260 0.5000 2.1240

H 2.1950 -1.4000 0.2170

O 2.7370 -2.1470 -0.0600

H 3.7170 -1.9440 -0.0170

O 0.7420 2.3310 0.5790

C	0.1030	3.6210	0.5640
H	0.5552	4.3389	1.2483
H	1.5440	2.2740	-0.0250
H	-0.9130	3.4590	0.8730
H	0.0820	4.0420	-0.4310
H	-2.2910	5.5580	-0.9030
O	-1.5290	6.1710	-0.8050
H	-0.9330	6.0000	-1.5450
H	-5.4910	-0.0898	2.1081
C	-4.6660	0.0520	1.4100
O	-5.2300	-0.0260	0.0730
H	-4.9940	0.7790	-0.4580
H	-4.2120	1.0230	1.5410
H	-3.9080	-0.7060	1.5360
H	2.8690	-1.8350	-2.0300
N	3.4950	-1.5280	-2.7530
C	3.2470	-0.4160	-3.4280
C	4.3970	0.0170	-4.3270
O	2.1940	0.2410	-3.3630
H	4.4510	-1.8580	-2.6470
H	4.3440	-0.5340	-5.2610
H	5.3410	-0.2080	-3.8490
H	4.3070	1.0730	-4.5390
O	5.2830	-1.5380	-0.0130
C	6.2250	-1.7030	-0.8420
C	7.6420	-1.5620	-0.2620
C	8.7280	-1.8760	-1.2930
O	6.1230	-1.9570	-2.0650
H	7.7040	-2.2190	0.5980
H	7.7380	-0.5480	0.1130
H	9.7220	-1.7530	-0.8720
H	8.6220	-1.2230	-2.1490
H	8.6210	-2.8920	-1.6500
H	1.6000	-3.5670	-0.1190
N	0.7450	-4.1070	-0.1950
C	0.3220	-4.8550	0.8200
C	-1.0870	-5.4010	0.6550
O	0.9660	-5.0590	1.8560
H	0.1650	-3.9630	-1.0010
H	-1.2940	-5.6580	-0.3740
H	-1.7730	-4.6150	0.9590
H	-1.2180	-6.2550	1.3030
N	2.8420	2.2190	-1.0950
C	4.0640	1.6380	-0.4800
C	4.1720	2.0400	0.9990
C	5.3980	1.4020	1.6790
C	5.3800	1.6300	3.2050
H	2.5580	1.7310	-1.9310
H	2.9170	3.2130	-1.2320
H	4.9660	1.9490	-1.0070
H	4.0100	0.5620	-0.5510
H	3.2670	1.7240	1.5050
H	4.2210	3.1220	1.0740
H	6.3050	1.8340	1.2590
H	5.4140	0.3430	1.4530
H	6.2410	1.1670	3.6780
H	4.4810	1.2080	3.6370
H	5.3990	2.6920	3.4340
H	-5.4970	-1.4260	-0.7080
O	-5.7820	-2.2460	-1.1960
H	-6.4360	-1.9800	-1.8560

Hydrolysis Transition State 1

RHF/6-31G*

C	-1.1410	0.1070	0.0500
N	-0.3930	0.6270	-1.0550
C	-0.7360	1.9410	-1.5380
C	0.4400	2.6410	-2.2770
C	1.2970	1.6980	-3.1250

C	1.6920	0.4970	-2.2730
C	0.4240	-0.2560	-1.8680
O	-1.5800	1.0080	0.8870
C	-1.9860	1.9350	-2.4120
N	-2.5020	3.1580	-2.6000
O	-2.5100	0.9070	-2.8690
N	2.3060	0.9600	-1.0110
O	3.6790	1.4270	-1.3440
S	4.1300	2.6030	-0.3510
O	3.2660	3.7450	-0.6310
O	5.5060	2.8110	-0.7630
O	3.9680	2.0710	0.9810
H	-0.9900	2.5330	-0.6790
H	-2.1060	3.9550	-2.1170
H	-3.3440	3.2490	-3.1260
H	0.0470	3.4480	-2.8790
H	1.0840	3.0580	-1.5320
H	0.7620	1.3410	-4.0000
H	2.1820	2.2300	-3.4550
H	2.3610	-0.1660	-2.8110
H	0.6730	-1.1090	-1.2670
H	-0.0770	-0.6030	-2.7610
H	2.4460	0.1880	-0.3700
O	-0.4370	-0.9930	0.6200
C	0.1210	-0.8490	1.9370
H	-0.5324	-1.3780	2.6308
H	0.1670	0.1930	2.2120
H	1.1100	-1.2720	1.9070
O	-2.2920	-0.6740	-0.6110
H	-2.5030	-0.2770	-1.4760
H	-3.1720	-1.3020	-0.0520
O	2.9350	-1.6450	0.3290
C	4.3010	-1.2970	0.0600
H	5.0212	-1.9564	0.5444
H	2.5340	-2.2880	-0.3190

H	4.4480	-0.3050	0.4480
H	4.5000	-1.2790	-1.0030
H	6.4500	1.3020	-1.1070
O	6.6010	0.4040	-1.4690
H	5.8210	0.2360	-2.0150
H	0.2505	3.8176	2.3585
C	0.7280	3.3040	1.5240
O	0.9100	4.2810	0.4590
H	1.8060	4.1720	0.0520
H	1.6960	2.9190	1.8120
H	0.1010	2.4920	1.1960
H	-2.4400	-1.1910	-3.5050
N	-2.7260	-2.1200	-3.2600
C	-1.8100	-3.0890	-3.1350
C	-2.3860	-4.4010	-2.6250
O	-0.6220	-2.9690	-3.4400
H	-3.6180	-2.2180	-2.8060
H	-2.7770	-4.9600	-3.4700
H	-3.1940	-4.2170	-1.9330
H	-1.6010	-4.9780	-2.1610
O	-4.1510	-1.7450	0.4500
C	-5.1410	-2.1250	-0.3130
C	-6.4980	-1.9760	0.3460
C	-7.6140	-2.6160	-0.4830
O	-5.0220	-2.5330	-1.4680
H	-6.6700	-0.9090	0.4780
H	-6.4320	-2.4090	1.3370
H	-8.5800	-2.4520	-0.0190
H	-7.4540	-3.6830	-0.5860
H	-7.6260	-2.1890	-1.4770
H	-4.4990	0.1120	1.0080
N	-4.3390	1.0970	0.9580
C	-5.3700	1.9190	0.8010
C	-4.9770	3.3820	0.6690
O	-6.5470	1.5440	0.7420

H	-3.3600	1.3550	0.9450
H	-4.4260	3.5170	-0.2570
H	-4.3280	3.6780	1.4830
H	-5.8690	3.9910	0.6520
N	1.7060	-3.5080	-1.2860
C	0.8440	-4.1930	-0.2900
C	1.6420	-4.5560	0.9730
C	0.7840	-5.3480	1.9790
C	1.4350	-5.4040	3.3760
H	1.1640	-3.2330	-2.0940
H	2.5150	-4.0510	-1.5410
H	0.3880	-5.0940	-0.6990
H	0.0600	-3.4960	-0.0290
H	2.0040	-3.6400	1.4190
H	2.5120	-5.1440	0.6960
H	0.6170	-6.3520	1.5990
H	-0.1890	-4.8750	2.0680
H	0.8820	-6.0650	4.0370
H	1.4440	-4.4140	3.8150
H	2.4550	-5.7710	3.3080
H	-0.3190	4.7480	-0.5110
O	-1.0680	5.1300	-1.0470
H	-0.7660	5.9610	-1.4360

Hydrolysis Intermediate

RHF/6-31G*

C	1.4060	0.0980	-0.0590
N	0.4790	0.4310	1.0000
C	0.7320	1.6590	1.7180
C	-0.4870	2.1390	2.5480
C	-1.2350	1.0050	3.2490
C	-1.5730	-0.0630	2.2130
C	-0.2730	-0.6350	1.6440
O	1.9520	1.1750	-0.5950
C	1.9620	1.5700	2.6110
N	2.4340	2.7740	2.9700
O	2.5010	0.5070	2.9550
N	-2.2800	0.5500	1.0720
O	-3.6420	0.9170	1.5490
S	-4.1910	2.1860	0.7340
O	-3.3780	3.3280	1.1350
O	-5.5590	2.2620	1.2120
O	-4.0510	1.8360	-0.6580
H	0.9620	2.4080	0.9850
H	2.0120	3.6150	2.5950
H	3.2480	2.8220	3.5440
H	-0.1530	2.8800	3.2610
H	-1.1770	2.6060	1.8770
H	-0.6350	0.5510	4.0330
H	-2.1440	1.3950	3.6900
H	-2.1590	-0.8630	2.6530
H	-0.4940	-1.3780	0.9000
H	0.2610	-1.1170	2.4480
H	-2.4470	-0.1390	0.3490
O	0.7710	-0.7950	-0.9850
C	-0.2770	-0.2410	-1.8100
H	0.1013	-0.1043	-2.8231
H	-0.6160	0.6990	-1.4100
H	-1.0980	-0.9360	-1.7920
O	2.4030	-0.8380	0.5230
H	2.5970	-0.5360	1.4290
H	3.4760	-1.5530	-0.1030
O	-2.8510	-1.8430	-0.5400
C	-4.2300	-1.6620	-0.1930
H	-4.9101	-2.2535	-0.8059
H	-2.3820	-2.5910	-0.0690
H	-4.4570	-0.6250	-0.3620
H	-4.4050	-1.8880	0.8510

H	-6.4300	0.6680	1.3280
O	-6.5170	-0.2810	1.5560
H	-5.7110	-0.4770	2.0520
H	-0.6861	4.1973	-1.8845
C	-0.8530	3.4710	-1.0890
O	-1.0880	4.2130	0.1390
H	-1.9700	3.9720	0.5210
H	-1.7200	2.8670	-1.3140
H	0.0100	2.8360	-0.9640
H	2.3290	-1.6820	3.3740
N	2.6680	-2.5780	3.0830
C	1.8110	-3.5660	2.8000
C	2.4770	-4.8150	2.2440
O	0.5960	-3.5150	3.0020
H	3.6000	-2.6040	2.7080
H	2.7800	-5.4470	3.0740
H	3.3530	-4.5600	1.6670
H	1.7630	-5.3560	1.6410
O	4.3960	-1.9030	-0.4870
C	5.3190	-2.3240	0.3530
C	6.7110	-2.2090	-0.2190
C	7.7440	-2.9430	0.6400
O	5.0970	-2.7430	1.4810
H	6.9330	-1.1450	-0.2760
H	6.6840	-2.5860	-1.2330
H	8.7430	-2.7970	0.2420
H	7.5350	-4.0060	0.6740
H	7.7150	-2.5670	1.6540
H	4.8060	0.0050	-1.0790
N	4.6540	0.9540	-0.8060
C	5.6770	1.6360	-0.3060
C	5.3130	3.0290	0.1860
O	6.8350	1.2050	-0.2370
H	3.6670	1.2110	-0.7210
H	4.4980	2.9720	0.8970
H	4.9800	3.6350	-0.6480
H	6.1800	3.4810	0.6450
N	-1.3560	-3.8670	0.4960
C	-0.3810	-4.0500	-0.6130
C	-1.1060	-4.1630	-1.9650
C	-0.1080	-4.2650	-3.1350
C	-0.7940	-4.0560	-4.5020
H	-0.8910	-3.7410	1.3840
H	-2.0490	-4.5960	0.5390
H	0.2470	-4.9290	-0.4630
H	0.2520	-3.1740	-0.6300
H	-1.7330	-3.2920	-2.0820
H	-1.7570	-5.0350	-1.9630
H	0.3860	-5.2330	-3.1080
H	0.6590	-3.5080	-3.0090
H	-0.1030	-4.2530	-5.3160
H	-1.1420	-3.0340	-4.5950
H	-1.6420	-4.7270	-4.6110
H	0.1710	4.5740	1.1390
O	0.8870	4.9040	1.7460
H	0.4800	5.5290	2.3610

Hydrolysis Transition State 2

RHF/6-31G*

C	1.7170	0.9020	0.4700
N	0.4970	1.0100	1.1980
C	0.2970	2.3010	1.8390
C	-1.1670	2.5140	2.2910
C	-1.7310	1.2950	3.0170
C	-1.5790	0.0860	2.1000
C	-0.0930	-0.1670	1.8300
O	2.0670	1.9320	-0.2030
C	1.1810	2.5210	3.0600
N	1.3590	3.8380	3.3020

O	1.6310	1.6410	3.7920
N	-2.2050	0.3700	0.7920
O	-3.6750	0.4290	1.0380
S	-4.3910	1.4380	0.0250
O	-3.9900	2.7850	0.4120
O	-5.7920	1.1620	0.2950
O	-3.9440	1.0590	-1.2940
H	0.5570	3.0510	1.1170
H	0.9610	4.5250	2.6820
H	1.8950	4.1180	4.0930
H	-1.2050	3.3950	2.9180
H	-1.7700	2.6890	1.4270
H	-1.1960	1.1020	3.9410
H	-2.7780	1.4570	3.2420
H	-2.0090	-0.8030	2.5490
H	0.0200	-1.0030	1.1620
H	0.3640	-0.4200	2.7690
H	-2.1080	-0.4350	0.1830
O	1.3750	-0.3620	-0.5920
C	0.3050	-0.2060	-1.5520
H	0.7288	-0.0065	-2.5362
H	-0.3060	0.6180	-1.2240
H	-0.2930	-1.1010	-1.5560
O	2.7590	0.2760	1.1600
H	2.7470	-0.6970	1.2000
H	2.2810	-0.9670	-0.9640
O	-2.1090	-2.1980	-0.4670
C	-3.5460	-2.2490	-0.4980
H	-3.9368	-3.0779	-1.0882
H	-1.6580	-2.7750	0.2120
H	-3.8640	-1.3320	-0.9600
H	-3.9620	-2.2850	0.4970
H	-6.3510	-0.5010	0.3570
O	-6.4960	-1.4480	0.5860
H	-6.0410	-1.5870	1.4270

H	-1.1603	4.0983	-2.2779
C	-1.2860	3.4200	-1.4340
O	-1.8780	4.1880	-0.3510
H	-2.7200	3.7590	-0.0560
H	-1.9420	2.6070	-1.7020
H	-0.3340	3.0260	-1.1120
H	2.1910	-0.2830	3.8100
N	2.6980	-1.1560	3.8240
C	2.0910	-2.3530	3.7740
C	3.0450	-3.5230	3.6150
O	0.8710	-2.5110	3.8290
H	3.6660	-1.0870	3.5980
H	3.8490	-3.4620	4.3410
H	3.4590	-3.4690	2.6160
H	2.4990	-4.4450	3.7460
O	3.2920	-1.6050	-1.3580
C	3.9610	-2.1990	-0.4250
C	5.3180	-2.7250	-0.8430
C	5.8820	-3.7350	0.1600
O	3.5550	-2.3070	0.7440
H	5.9660	-1.8560	-0.9100
H	5.2190	-3.1470	-1.8340
H	6.8710	-4.0650	-0.1400
H	5.2370	-4.6030	0.2400
H	5.9500	-3.2790	1.1380
H	4.5070	0.4160	-1.8000
N	4.6200	1.1360	-1.1160
C	5.6910	1.0640	-0.3140
C	5.5940	1.9680	0.8990
O	6.6570	0.3230	-0.5130
H	3.7620	1.5720	-0.7880
H	4.7380	1.6400	1.4770
H	5.4210	2.9930	0.5940
H	6.5030	1.8970	1.4770
N	-0.5800	-3.7810	1.1730

C	0.5720	-4.1270	0.2950
C	0.1080	-4.3500	-1.1570
C	1.2810	-4.4200	-2.1550
C	0.7810	-4.3870	-3.6160
H	-0.2750	-3.4230	2.0670
H	-1.2370	-4.5370	1.2760
H	1.1040	-5.0100	0.6500
H	1.2640	-3.3000	0.3350
H	-0.5460	-3.5360	-1.4250
H	-0.4790	-5.2650	-1.2140
H	1.8610	-5.3230	-1.9820
H	1.9360	-3.5730	-1.9940
H	1.6050	-4.5240	-4.3100
H	0.3120	-3.4340	-3.8370
H	0.0560	-5.1780	-3.7930
H	-0.8440	4.9020	0.7530
O	-0.2860	5.4480	1.3660
H	-0.8780	6.0650	1.8180

Hydrolysis Products

RHF/6-31G*

C	0.8620	2.0050	0.8720
N	0.1040	1.2740	1.7330
C	-0.9600	1.9800	2.4310
C	-2.1850	1.1030	2.7760
C	-1.7880	-0.3030	3.2150
C	-0.9360	-0.9200	2.1130
C	0.3740	-0.1430	1.9900
O	0.4540	3.0410	0.3330
C	-0.4150	2.5990	3.7140
N	-1.2090	3.5790	4.1820
O	0.6210	2.2460	4.2770
N	-1.6240	-0.8120	0.8110
O	-2.8610	-1.6390	0.9590
S	-4.0260	-1.1450	-0.0110
O	-4.5210	0.1160	0.5300
O	-4.9900	-2.2220	0.1250
O	-3.4280	-1.0220	-1.3180
H	-1.2840	2.7830	1.7950
H	-2.0300	3.8690	3.6650
H	-0.9460	4.0460	5.0230
H	-2.7510	1.6040	3.5470
H	-2.8020	1.0160	1.9060
H	-1.2110	-0.2850	4.1350
H	-2.6770	-0.8980	3.3720
H	-0.6970	-1.9530	2.3370
H	0.9370	-0.5330	1.1600
H	0.9150	-0.2950	2.9040
H	-1.0950	-1.3200	0.1100
O	0.7040	0.0030	-1.1360
C	-0.0430	-0.3830	-2.2960
H	0.1337	0.3449	-3.0879
H	-1.0850	-0.3930	-2.0180
H	0.2320	-1.3700	-2.6400
O	2.1190	1.6630	0.6680
H	2.6050	0.8010	0.8000
H	1.6760	-0.0230	-1.3210
O	-0.2680	-3.0080	-0.4860
C	-1.4530	-3.8160	-0.5510
H	-1.2966	-4.7623	-1.0688
H	0.3420	-3.1970	0.2740
H	-2.1720	-3.2410	-1.1080
H	-1.8690	-4.0040	0.4260
H	-4.6500	-3.9110	0.2280
O	-4.3660	-4.8250	0.4610
H	-4.0010	-4.7800	1.3540
H	-3.4259	3.0259	-1.6926
C	-2.8880	2.5800	-0.8560
O	-3.7530	2.6100	0.3110
H	-4.1520	1.7110	0.4290

H	-2.6390	1.5570	-1.0910
H	-1.9820	3.1210	-0.6330
H	2.2100	1.1230	3.9410
N	3.1200	0.7400	3.7330
C	3.3690	-0.5820	3.7490
C	4.7740	-0.9540	3.3250
O	2.5260	-1.4300	4.0460
H	3.7790	1.3620	3.3190
H	5.5070	-0.3540	3.8510
H	4.8460	-0.7610	2.2600
H	4.9390	-2.0020	3.5250
O	3.3150	0.1210	-1.7190
C	4.1680	0.1510	-0.7910
C	5.5920	0.5120	-1.2080
C	6.5960	0.4410	-0.0560
O	3.9110	-0.0660	0.4330
H	5.5480	1.5130	-1.6280
H	5.8690	-0.1550	-2.0170
H	7.5870	0.7350	-0.3880
H	6.6500	-0.5640	0.3450
H	6.2890	1.1000	0.7440
H	2.9610	2.1810	-1.8890
N	2.7850	3.1550	-1.7110
C	3.7780	4.0380	-1.8630
C	3.4300	5.4390	-1.3800
O	4.8780	3.7770	-2.3460
H	2.0090	3.3350	-1.0980
H	3.1800	5.4110	-0.3250
H	2.5660	5.8120	-1.9170
H	4.2770	6.0900	-1.5410
N	1.7090	-3.4710	1.4370
C	2.9420	-3.2960	0.6190
C	2.7740	-3.9180	-0.7790
C	3.8820	-3.4600	-1.7470
C	3.7950	-4.1950	-3.1000

H	1.7740	-2.9580	2.3040
H	1.4810	-4.4390	1.5950
H	3.8150	-3.7350	1.1040
H	3.1190	-2.2360	0.5280
H	1.8140	-3.6140	-1.1660
H	2.7740	-5.0030	-0.6960
H	4.8590	-3.6350	-1.3040
H	3.7740	-2.3950	-1.9110
H	4.5630	-3.8490	-3.7840
H	2.8290	-4.0170	-3.5610
H	3.9140	-5.2670	-2.9640
H	-3.5360	3.5360	1.6930
O	-3.5450	3.9690	2.5870
H	-4.2840	3.5830	3.0760

3. Supplementary References

1 S. D. Lahiri, S. Mangani, T. Durand-Reville, M. Benvenuti, F. D. Luca, G. Sanyal, J.-D. Docquier, *Antimicrob. Agents Chemother.,* 2013, **57**, 2496.

2 W. Ke, C. R. Bethel, J. M. Thomson, R. A. Bonomo, F. van den Akker, *Biochemistry,* 2007, **46**, 5732.

3 D. E. Ehmann, H. Jahić, P. L. Ross, R.-F. Gu, J. Hu, T. F. Durand-Réville, S. Lahiri, J. Thresher, S. Livchak, N. Gao, T. Palmer, G. K. Walkup, S. L. Fisher, *J. Biol. Chem.,* 2013, **288**, 27960.

4 M. W. Schmidt, K. K. Baldridge, J. A. Boatz, S. T. Elbert, M. S. Gordon, J. H. Jensen, S. Koseki, N. Matsunaga, K. A. Nguyen, S. J. Su, T. L. Windus, M. Dupuis, J. A. Montgomery, *J. Comp. Chem.,* 1993, **14**, 1347.

5 C. Gonzalez, B. H. Schlegel, *J. Chem. Phys.,* 1989, **90**, 2154.

6 A. D. Becke, *Phys. Rev. A*, 1988, **38**, 3098.
